# National burden of cancer in Italy, 1990–2017: a systematic analysis for the global burden of disease study 2017

**DOI:** 10.1038/s41598-020-79176-3

**Published:** 2020-12-16

**Authors:** Cristina Bosetti, Eugenio Traini, Tahiya Alam, Christine A. Allen, Giulia Carreras, Kelly Compton, Christina Fitzmaurice, Lisa M. Force, Silvano Gallus, Giuseppe Gorini, James D. Harvey, Jonathan M. Kocarnik, Carlo La Vecchia, Alessandra Lugo, Mohsen Naghavi, Alyssa Pennini, Cristiano Piccinelli, Luca Ronfani, Rixing Xu, Lorenzo Monasta

**Affiliations:** 1 Department of Oncology, Istituto di Ricerche Farmacologiche Mario Negri IRCCS, Via Mario Negri, 2, 20156 Milan, Italy; 2 Clinical Epidemiology and Public Health Research Unit, Institute for Maternal and Child Health IRCCS Burlo Garofolo, Trieste, Italy; 3 Institute for Health Metrics and Evaluation, University of Washington, Seattle, WA USA; 4 Oncologic Network, Prevention, and Research Institute, Florence, Italy; 5 Division of Hematology, University of Washington, Seattle, WA USA; 6 Department of Oncology, St. Jude Children’s Research Hospital, Memphis, TN USA; 7 Department of Global Pediatric Medicine, St. Jude Children’s Research Hospital, Memphis, TN USA; 8 Department of Environmental Health Sciences, Istituto Di Ricerche Farmacologiche Mario Negri IRCCS, Milan, Italy; 9 Public Health Sciences Division, Fred Hutchinson Cancer Research Center, Seattle, WA USA; 10 Department of Clinical Sciences and Community Health, University of Milan, Milan, Italy; 11 Department of Health Metrics Sciences, School of Medicine, University of Washington, Seattle, WA USA; 12 CPO Piemonte, AOU Città Della Salute E Della Scienza, Torino, Italy

**Keywords:** Cancer epidemiology, Epidemiology

## Abstract

We monitored the burden of cancer in Italy and its trends over the last three decades, providing estimates of cancer incidence, mortality, years of life lost, years lived with disability, and disability-adjusted life-years (DALYs), for cancer overall and 30 cancer sites using data from the Global Burden of Disease study 2017. An overview of mortality trends between 1990 and 2017 was also provided. In 2017, there were 254,336 new cancer cases in men and 214,994 in women, corresponding to an age-standardized incidence rate (ASIR) of 438 and 330/100,000, respectively. Between 1990 and 2017, incident cancer cases, and, to a lesser extent, ASIRs significantly increased overall and for almost all cancer sites, but ASIRs significantly declined for lung and other tobacco-related neoplasms. In 2017, there were 101,659 cancer deaths in men (age-standardized death rate, ASDR, 158.5/100,000) and 78,918 in women (ASDR 93.9/100,000). Cancer deaths significantly increased between 1990 and 2017 (+ 18%), but ASDR significantly decreased (− 28%). Deaths significantly increased for many cancer sites, but decreased for stomach, esophageal, laryngeal, Hodgkin lymphoma, and testicular cancer. ASDRs significantly decreased for most neoplasms, with the main exceptions of cancer of the pancreas and uterus, and multiple myeloma. In 2017, cancer caused 3,204,000 DALYs. Between 1990 and 2017, DALYs and age-standardized DALY rates significantly declined (-3.4% and -33%, respectively). Age-standardized mortality rates in Italy showed favorable patterns over the last few decades. However, the absolute number of cancer cases and, to a lower extent, of cancer deaths increased likely due to the progressive ageing of the population, this calling for a continuous effort in cancer prevention, early diagnosis, and treatment.

## Introduction

Neoplasms represent the second cause of death following cardiovascular diseases, with 9.6 million deaths and 233.5 million disability-adjusted life-years (DALYs) worldwide in 2017^1,2^. Cancer cases has been increasing over the last decades (by 33% between 2007 and 2017, reaching 24.5 million new cancer cases in 2017), largely due to population ageing and growth^1,2^. While age-standardized incidence rates remained relatively stable since 1990, age-standardized death rates have been decreasing globally over the last decades, due to improvements in cancer diagnosis and treatment, as well as to the control of some risk factors, such as tobacco and alcohol^1,3^. However, a large variability in incidence and mortality cancer rates still exists, particularly between high- and low-income countries ^1,2^, due to different socio-demographic level, education, prevention strategies, and access to effective treatments and specialized care^4^.

In Italy, according to the National Institute of Statistics and the Global Cancer Observatory, there are about 410,000 new cancer cases and 175,000 cancer deaths each year^5,6^. Overall cancer mortality has been declining in both sexes over the last decades, with favorable trends for most common cancer sites^7,8^. Data on cancer incidence and other indicators of cancer burden in Italy are scanty. A continuous monitoring of the burden of cancer is of outmost importance in order to plan proper allocation of health resources for cancer prevention, diagnosis, and management.

In this regard, using the Global Burden of Disease (GBD) 2017 study, we provide a specific focus on the burden of cancer in Italy, including information on cancer incidence, mortality, years of life lost (YLLs), years lived with disability (YLDs), and DALYs. An overview of mortality trends between 1990 and 2017, as well as a comparison of incidence, mortality, and DALYs with other countries of Western Europe is also provided.

## Results

### Incidence

In 2017, in Italy there were 254,336 new cancer cases (95% UI 237,798–279,776) in men and 214,994 (199,575–230,541) in women, corresponding to an age-standardized incidence rate (ASIR) per 100,000 of 438 (95% UI 409–480) and 330 (95% UI 306–356), respectively (Table 1). Cancer of non-melanoma skin (43,003), prostate (40,927), trachea, bronchus, and lung (TBL; 28,651), and colorectum (28,602) were the most frequent sites in men, accounting for 56% of all male cancers. For women, the most common incident cancers were breast (49,500), non-melanoma skin (31,080), colorectal (23,626), and TBL (12,325) cancer, these representing 54% of all female cancer cases.

**Table 1:** Incidence, deaths, and corresponding age-standardized rates for all cancers and 30 cancer groups. Italy, 2017.

Cancer site	Number of incident cases (95% UI)			Age-standardized incidence rate per 100 000 person-years (95% UI)		Number of deaths (95% UI)			**Age-standardized death rate per 100 000 person-years (95% UI)**	
	Total	Male	Female	Male	Female	Total	Male	Female	**Male**	**Female**
All cancers^a^	469,330 (444,652 – 497,521)	254,336 (237,798 – 279,776)	214,994 (199,575 – 230,541)	438.0 (408.6 – 479.6)	330.4 (305.5 – 355.6)	180,577 (170,974 – 189,903)	101,659 (92,220 – 108,583)	78,918 (72,831 – 84,942)	158.5 (148.4 – 169.6)	93.9 (86.4 – 101.4)
Lip and oral cavity cancer	4864 (4402 – 5366)	2899 (2524 – 3316)	1965 (1669 – 2289)	5.1 (4.5 – 5.9)	2.7 (2.3 – 3.1)	1878 (1744 – 2024)	1164 (1046 – 1291)	714 (630 – 800)	1.9 (1.7 – 2.1)	0.8 (0.7 – 0.9)
Nasopharynx cancer	1108 (909 – 1329)	933 (740 – 1151)	174 (131 – 216)	1.8 (1.4 – 2.3)	0.4 (0.3 – 0.4)	638 (579 – 714)	556 (497 – 630)	83 (72 – 94)	0.9 (0.8 – 1.0)	0.1 (0.1 – 0.2)
Other pharynx cancer	2113 (1894 – 2366)	1614 (1408 – 1834)	499 (417 – 588)	3.0 (2.6 – 3.4)	0.9 (0.7 – 1.0)	873 (797 – 968)	648 (574 – 730)	225 (200 – 255)	1.1 (1.0 – 1.3)	0.3 (0.3 – 0.4)
Esophageal cancer	2468 (2242 – 2728)	1707 (1516 – 1933)	760 (657 – 883)	2.9 (2.6 – 3.3)	0.9 (0.8 – 1.0)	2168 (1999 – 2347)	1546 (1394 – 1716)	622 (560 – 685)	2.5 (2.2 – 2.8)	0.7 (0.6 – 0.7)
Stomach cancer	18,639 (16,544 – 20,965)	11,145 (9434 – 12,960)	7494 (6298 – 8948)	17.7 (15.1 – 20.4)	8.7 (7.4 – 10.3)	12,117 (11,267 – 12,996)	7011 (6373 – 7696)	5107 (4573 – 5676)	10.7 (9.8 – 11.7)	5.4 (4.8 – 6.0)
Colon and rectum cancer	52,228 (48,427 – 56,835)	28,602 (25,230 – 32,134)	23,626 (21,087 – 26,516)	46.5 (41.1 – 52.2)	29.8 (26.5 – 33.2)	20,982 (19,498 – 22,621)	11,172 (9994 – 12,454)	9810 (8866 – 10,943)	17.1 (15.2 – 18.9)	10.5 (9.4 – 11.6)
Liver cancer	12,520 (11,015 – 14,183)	8413 (7158 – 9836)	4107 (3441 – 5065)	14.0 (11.9 – 16.3)	4.9 (4.2 – 5.8)	10,616 (9617 – 11,717)	6905 (6111 – 7860)	3711 (3259 – 4185)	10.9 (9.6 – 12.4)	4.0 (3.5 – 4.5)
Gallbladder and biliary tract cancer	5728 (4698 – 7326)	2626 (1832 – 3478)	3101 (2423 – 4534)	4.1 (2.9 – 5.4)	3.5 (2.8 – 4.6)	4307 (3778 – 4718)	1971 (1375 – 2248)	2336 (2077 – 2614)	3.0 (2.1 – 3.4)	2.5 (2.2 – 2.8)
Pancreatic cancer	13,475 (12,189 – 14,925)	6224 (5468 – 7167)	7251 (6209 – 8530)	9.9 (8.8 – 11.3)	8.3 (7.2 – 9.6)	12,681 (11,784 – 13,695)	5903 (5352 – 6615)	6778 (6089 – 7498)	9.3 (8.4 – 10.5)	7.5 (6.7 – 8.3)
Larynx cancer	4509 (4017 – 5113)	4119 (3631 – 4699)	390 (330 – 460)	7.2 (6.3 – 8.2)	0.6 (0.5 – 0.7)	1595 (1459 – 1741)	1433 (1296 – 1574)	162 (142 – 181)	2.3 (2.1 – 2.5)	0.2 (0.2 – 0.2)
Tracheal, bronchus, and lung cancer	40,977 (37,242 – 45,194)	28,651 (25,099 – 32,514)	12,325 (10,754 – 13,947)	45.4 (40.0 – 51.3)	16.9 (14.7 – 19.0)	34,099 (31,925 – 36,342)	24,438 (22,416 – 26,732)	9661 (8619 – 10,735)	37.9 (34.9 – 41.4)	12.2 (10.9 – 13.6)
Malignant skin melanoma	13,492 (8313 – 15,890)	5951 (2497 – 7719)	7542 (4547 – 9433)	12.4 (5.3 – 16.1)	15.7 (9.2 – 19.7)	2096 (1290 – 2397)	1045 (414 – 1298)	1051 (657 – 1338)	1.8 (0.7 – 2.3)	1.4 (0.9 – 1.8)
Non-melanoma skin cancer	74,083 (71,457 – 76,972)	43,003 (41,119 – 44,616)	31,080 (29,739 – 32,578)	71.2 (68.2 – 73.8)	44.4 (42.6 – 46.4)	1189 (1103 – 1293)	817 (741 – 912)	372 (332 – 410)	1.2 (1.1 – 1.4)	0.3 (0.3 – 0.3)
Breast cancer	50,106 (44,734 – 55,559)	605 (533 – 686)	49,500 (44,121 – 55,000)	1.0 (0.9 – 1.1)	83.3 (74.3 – 93.1)	13,013 (11,800 – 14,174)	244 (217 – 278)	12,769 (11,548 – 13,928)	0.4 (0.3 – 0.4)	16.5 (14.8 – 18.0)
Cervical cancer	3118 (2708 – 3576)	0 (0 – 0)	3118 (2708 – 3576)	0.0 (0.0 – 0.0)	6.4 (5.5 – 7.5)	1622 (1455 – 1798)	0 (0 – 0)	1622 (1455 – 1798)	0.0 (0.0 – 0.0)	2.2 (1.9 – 2.4)
Uterine cancer	14,650 (12,654 – 17,029)	0 (0 – 0)	14,650 (12,654 – 17,029)	0.0 (0.0 – 0.0)	23.1 (19.8 – 27)	1604 (1438 – 1798)	0 (0 – 0)	1604 (1438 – 1798)	0.0 (0.0 – 0.0)	1.9 (1.7 – 2.2)
Ovarian cancer	5413 (4807 – 6098)	0 (0 – 0)	5413 (4807 – 6098)	0.0 (0.0 – 0.0)	9.2 (8.1 – 10.4)	3838 (3438 – 4271)	0 (0 – 0)	3838 (3438 – 4271)	0.0 (0.0 – 0.0)	5.1 (4.5 – 5.7)
Prostate cancer	40,927 (33,019 – 65,561)	40,927 (33,019 – 65,561)	0 (0 – 0)	65.5 (52.5 – 105.3)	0.0 (0.0 – 0.0)	9713 (8177 – 14,235)	9713 (8177 – 14,235)	0 (0 – 0)	13.7 (11.6 – 20.2)	0.0 (0.0 – 0.0)
Testicular cancer	2405 (1915 – 2922)	2405 (1915 – 2922)	0 (0 – 0)	8.6 (6.8 – 10.7)	0.0 (0.0 – 0.0)	94 (82 – 107)	94 (82 – 107)	0 (0 – 0)	0.2 (0.2 – 0.3)	0.0 (0.0 – 0.0)
Kidney cancer	10,742 (9244 – 12,082)	7043 (6058 – 8029)	3698 (2361 – 4270)	12.6 (10.8 – 14.4)	5.7 (3.7 – 6.7)	4311 (3659 – 4755)	2843 (2566 – 3166)	1468 (899 – 1687)	4.5 (4.0 – 5.0)	1.7 (1.0 – 1.9)
Bladder cancer	23,427 (21,341 – 25,710)	18,909 (16,932 – 21,138)	4518 (4039 – 5063)	29.8 (26.7 – 33.4)	5.1 (4.6 – 5.8)	7634 (6972 – 8357)	5995 (5395 – 6705)	1639 (1455 – 1828)	8.7 (7.8 – 9.7)	1.5 (1.3 – 1.7)
Brain and nervous system cancer	9100 (6631 – 11,192)	5314 (3414 – 6996)	3787 (2805 – 4924)	12.1 (8.2 – 15.9)	8.0 (6.1 – 10.3)	4036 (2864 – 4531)	2234 (1461 – 2627)	1802 (1302 – 2063)	4.3 (2.9 – 5.1)	2.9 (2.3 – 3.4)
Thyroid cancer	6737 (5844 – 7639)	2361 (1911 – 2905)	4376 (3684 – 5134)	4.7 (3.8 – 5.9)	8.8 (7.3 – 10.5)	631 (569 – 689)	269 (235 – 306)	362 (317 – 405)	0.4 (0.4 – 0.5)	0.4 (0.4 – 0.5)
Mesothelioma	1746 (1555 – 1955)	1267 (1098 – 1476)	479 (422 – 539)	2.0 (1.8 – 2.4)	0.6 (0.6 – 0.7)	1714 (1557 – 1901)	1229 (1096 – 1386)	485 (431 – 543)	1.9 (1.7 – 2.1)	0.6 (0.5 – 0.6)
Hodgkin lymphoma	2626 (2095 – 3544)	1528 (1101 – 2369)	1098 (841 – 1457)	4.8 (3.3 – 7.5)	3.7 (2.7 – 5.2)	445 (359 – 574)	268 (195 – 375)	177 (144 – 218)	0.5 (0.4 – 0.8)	0.3 (0.2 – 0.4)
Non-Hodgkin lymphoma	15,012 (13,453 – 16,624)	8263 (7062 – 9575)	6749 (5869 – 7679)	16.3 (14.0 – 18.7)	11.0 (9.6 – 12.5)	5376 (4956 – 5813)	2975 (2652 – 3321)	2400 (2120 – 2690)	4.8 (4.3 – 5.4)	2.9 (2.5 – 3.2)
Multiple myeloma	5983 (5094 – 7461)	3270 (2454 – 4306)	2713 (2206 – 3822)	5.4 (4.1 – 7.3)	3.5 (2.8 – 5.0)	3638 (3187 – 4413)	1875 (1414 – 2394)	1763 (1486 – 2368)	2.8 (2.2 – 3.7)	1.9 (1.6 – 2.7)
Leukemia	16,151 (14,730 – 17,585)	9390 (8337 – 10,569)	6761 (5878 – 7681)	18.2 (16.1 – 20.4)	10.7 (9.3 – 12.4)	7631 (7070 – 8207)	4377 (3961 – 4805)	3255 (2874 – 3643)	7.2 (6.6 – 7.9)	4.0 (3.6 – 4.5)
Other malignants neoplasms^b^	14,983 (12,323 – 17,157)	7166 (5309 – 9047)	7818 (5987 – 9181)	15.9 (12.1 – 20.0)	13.6 (10.5 – 16.1)	5332 (4401 – 5844)	2670 (2192 – 3055)	2662 (2063 – 2999)	4.7 (3.8 – 5.4)	3.4 (2.6 – 3.8)
Other neoplasms^c^	197,824 (191,329 – 204,430)	84,896 (81,676 – 88,555)	112,928 (108,946 – 117,096)	182.5 (176.7 – 189.0)	252.3 (244.0 – 261.0)	4705 (3114 – 6362)	2263 (985 – 3659)	2441 (1539 – 3549)	3.5 (1.5 – 5.8)	2.7 (1.8 – 4.0)

UI: uncertainty intervals.

^a^Includes malignant neoplasms (International Classification of Diseases 10 [ICD-10] codes C00-C96, excluding Kaposi sarcoma, ICD-10 C46), benign/in situ neoplasms (ICD-10 D00-D49), and other malignant neoplasms (ICD-10 codes C17, C30-C31, C37, C38, C40-C41, C47-C49, C4A, C51-C52, C57-C58, C60, C63, C66, C68, C69, C74-C75). ^b^Includes other malignant neoplasms (ICD-10 codes C17, C30-C31, C37, C38, C40-C41, C47-C49, C4A, C51-C52, C57-C58, C60, C63, C66, C68, C69, C74-C75). ^c^Includes benign/in situ neoplasms (ICD-10 D00-D49).

Between 1990 and 2017, incident cancer cases, incidence rate, and, to a lesser extent, ASIR significantly increased in both sexes combined (+ 61%, + 50%, and + 9%; Supplementary Table S1). Cancer cases and incidence rates significantly increased for almost all cancer sites, the only exceptions being stomach and laryngeal cancer, while ASIRs significantly increased for several cancers, but significantly declined for TBL, bladder, stomach, ovarian, lip and oral, laryngeal, and esophageal cancer.

### Mortality

In 2017, there were 101,659 (95% UI 92,220–108,583) cancer deaths in men and 78,918 (95% UI 72,831–84,942) in women, corresponding to an age-standardized death rate (ASDR) per 100,000 of 158.5 (95% UI 148.4–169.6) in men and of 93.9 (95% UI 86.4 – 101.4) in women (Table 1). In men, the most common causes of cancer deaths were TBL, colorectum, prostate, and stomach cancer, with 24,438, 11,172, 9713, and 7011 deaths, respectively; in women, the major causes of cancer deaths were breast, colorectal, TBL, and pancreatic cancer, with 12,769, 9,810, 9,661, 6,778 deaths, respectively.

While cancer deaths and death rate significantly increased between 1990 and 2017 by 18% and 11%, respectively, ASDR significantly decreased by 28% (Supplementary Table S2). Deaths and death rates in both sexes combined significantly increased for many cancer sites, but significantly decreased for stomach, esophageal, laryngeal, Hodgkin lymphoma, and testicular cancer. ASDRs significantly decreased for most neoplasms, with the exceptions of cancer of the pancreas, multiple myeloma, and uterus.

### DALYs, YLLs and YLDs

In 2017, cancer caused 3,204,000 (95% UI 3,018,000–3,396,000) DALYs in Italy, of which 94% came from YLLs (3,012,000) and 6% from YLDs (192,000; Supplementary Tables S3–S6). The proportion of DALYs due to YLLs was the highest (≥ 98%) for cancer of the TBL, pancreas, liver, gallbladder, and esophagus, while was the lowest (< 85%) for cancer of the prostate, uterus, and thyroid (Supplementary Table S3).

The leading causes of cancer DALYs and YLLs for both sexes combined in 2017 were TBL (612,000 and 602,000, respectively), colon and rectum (345,000 and 319,000), breast (276,000 and 240,000), and pancreatic (211,000 and 208,000) cancer (Supplementary Tables S4–S5). The leading causes of cancer YLDs were breast (36,200), colon and rectum (26,600), prostate (26,300), and bladder (12,300) cancer (Supplementary Table S6).

Between 1990 and 2017, a decline in DALYs (− 3.4%) and DALY rates (− 9.4%) was observed (Supplementary Table S4). DALYs and DALY rates significantly decreased for several cancer sites, including TBL, stomach, and leukemia, although they significantly increased for other cancer sites, such as pancreatic, prostate, kidney, multiple myeloma, uterine, cervical, and non-melanoma skin cancer. Age-standardized DALY rates declined overall (-33%) and for most cancer sites.

The patterns for YLLs, YLL rates, and age-standardized YLL rates were consistent with those for DALYs, DALY rates, and age-standardized DALY rates, with declines by − 6%, − 12% and − 34% for all cancers combined (Supplementary Table S5).

Overall and for all cancer sites, YLDs and YLD rates significantly increased between 1990 and 2017 (by 68% and 58%, respectively, for all cancers; Supplementary Table S6). Similarly, age-standardized YLD rates significantly increased overall (+ 20%) and for many cancer sites (including the most frequent ones), although they significantly decreased for some other cancers, such as bladder, stomach, and larynx.

### Trends in mortality rates and ASDRs over time

**Figure 1:**
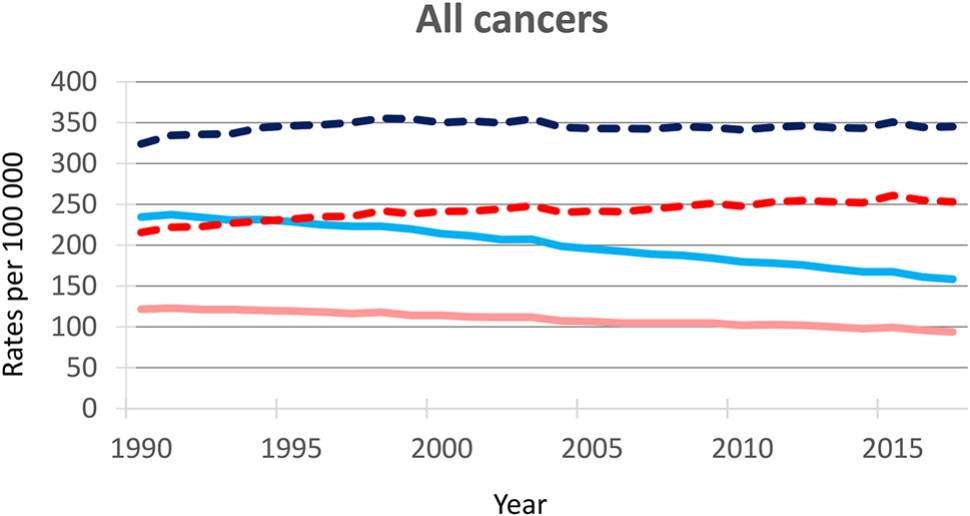
Trends in age-standardized and all ages death rates from all cancers^a^, Italy, 1990–2017. *Legend:* Solid lines show age-standardized death rates for men (light blue) and women (light red), dashed lines show all ages death rates for men (blue) and women (red). ^a^Includes malignant neoplasms (International Classification of Diseases 10 [ICD-10] codes C00-C96, excluding Kaposi sarcoma, ICD-10 C46), benign/in situ neoplasms (ICD-10 D00-D49), and other malignant neoplasms (ICD-10 codes C17, C30-C31, C37, C38, C40-C41, C47-C49, C4A, C51-C52, C57-C58, C60, C63, C66, C68, C69, C74-C75).

Figure 1 shows the trends in death rates and ASDRs for all cancers over the period 1990 and 2017, for men and women separately. Cancer ASDRs declined over the last decades in both men and women, while death rates levelled-off since the mid 2000s in men and since early 2010s in women (Fig. 1).

**Figure 2:**
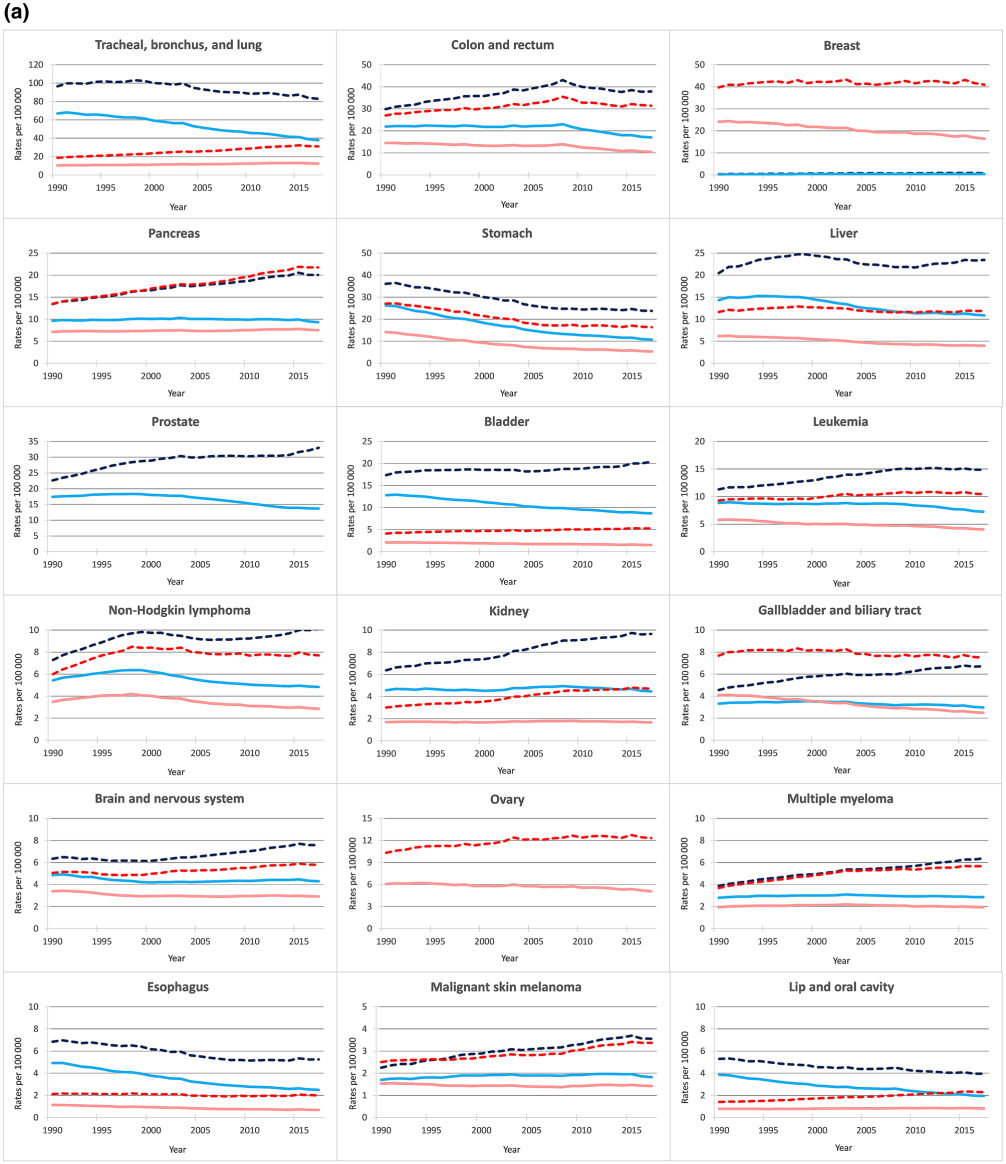

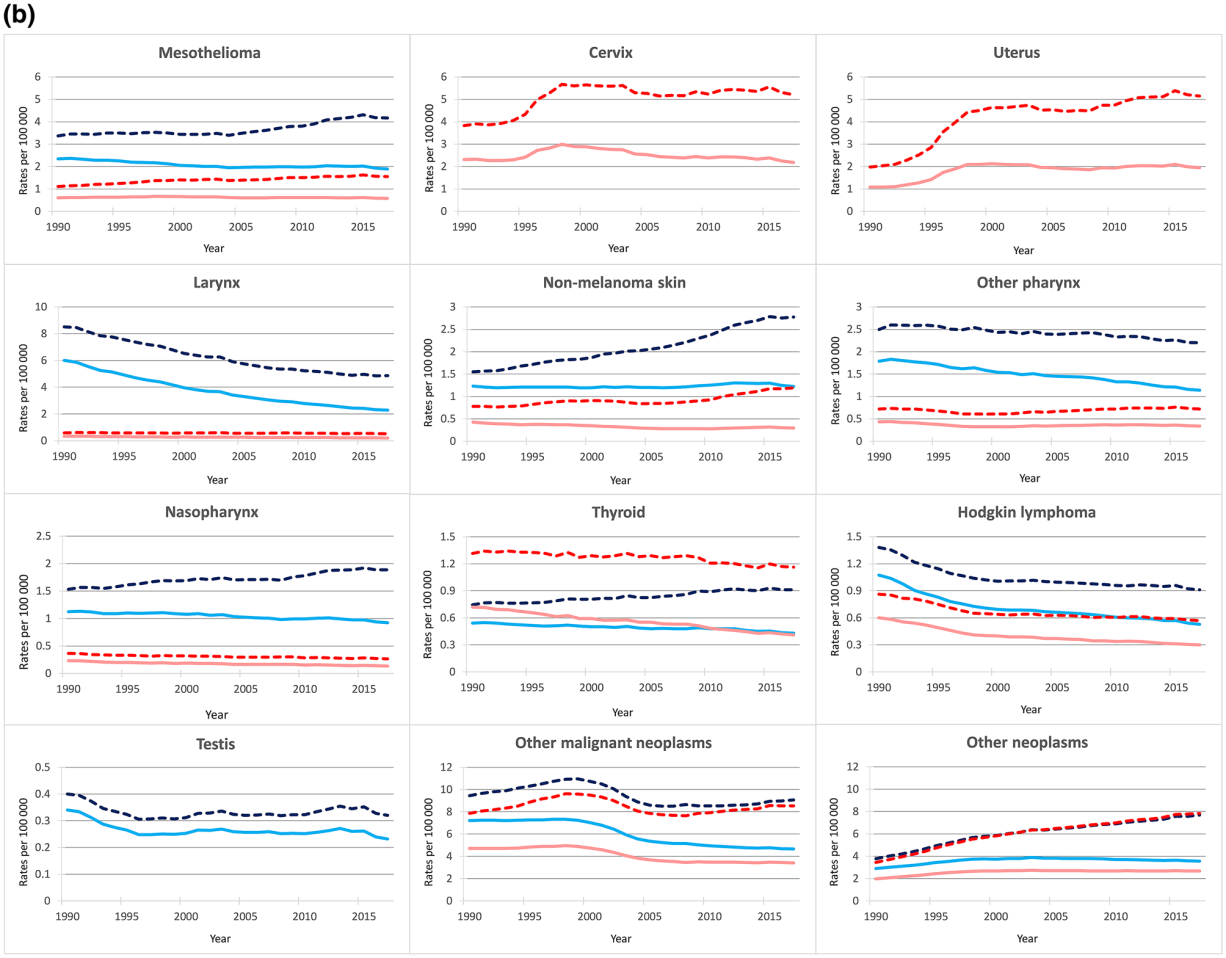
Trends in age-standardized and all ages death rates for 30 cancer groups (ordered by ranking in number of deaths in 2017), Italy, 1990–2017. (**a**) Cancer of the: trachea, bronchus, and lung; colon and rectum; breast; pancreas; stomach; liver; prostate; bladder; leukemia; non-Hodgkin lymphoma; kidney; gallbladder and biliary tract; brain and nervous system; ovary; multiple myeloma; esophagus; malignant skin melanoma; and lip and oral cavity. (**b**) Cancer of the: mesothelioma; cervix, uterus; larynx; non-melanoma skin; other pharynx; nasopharynx; thyroid; Hodgkin lymphoma; testis; other malignant neoplasms; and other neoplasms. *Legend:* Solid lines show age-standardized death rates for men (light blue) and women (light red), dashed lines show all ages death rates for men (blue) and women (red).

Figures 2a and b give the corresponding trends for the 30 cancer categories. Death rates increased over the last decades for several neoplasms, including female TBL, breast, pancreas, prostate, bladder, and kidney. Death rates were stable, at least over more recent calendar years, for cancer of the liver (since late 1990s), leukemia (since mid 2000s), non-Hodgkin lymphoma (since 2005), female gallbladder, cervix (since mid 1990s), and female upper aerodigestive tract cancers. Declines in death rates were observed for male TBL, male upper aerodigestive tract cancers, colorectum (since the late 1990s), stomach, female thyroid, Hodgkin lymphoma, and testicular cancer.

ASDRs declined for most cancers, with long-term reductions for TBL, male upper aerodigestive tract, male bladder, female gallbladder, breast, stomach, prostate, ovarian, mesothelioma, non-melanoma skin, thyroid and Hodgkin lymphoma, and more recent declines for colorectum (since the late 2000s), liver (since the late 1990s), leukemia (since the early 2000s), non-Hodgkin lymphoma (since the late 1990s), and cervix cancer (since the late 1990s; Figs. 2a and b). ASDRs were stable over the last decades for female TBL, other female upper aerodigestive tract cancers, female bladder cancer, kidney, male gallbladder, brain, multiple myeloma, melanoma, female mesothelioma, and uterus (since the late 1990′s). Only for pancreatic cancer, ASDRs non significantly increased up to more recent calendar years, even if a levelling-off was observed since 2015.

### Comparisons with Western European countries

In 2017, Italy had lower age-standardized incidence (375.5), death (121.2), and DALY (2676.6) rates compared to Western Europe overall (407.7, 128.8, and 2877.7, respectively; Table 2). In particular, these rates were lower than those of the other most populous Western European countries (i.e., France, Germany, and the United Kingdom), with the only exception of Spain. Moreover, between 1990 and 2017 ASIR increased by a lower extent in Italy (+ 9.4%) than in Western Europe (+ 13.7%) and other Western European countries, while ASDRs and age-standardized DALY rates decreased more strongly in Italy (− 28.2% and − 32.5%) than in Western Europe (− 22.4% and − 25.6%) and the other Western European countries.

**Table 2:** Age-standardized incidence, death, and disability adjusted life years (DALY) rates for all cancer ^a^ in Western Europe and selected countries of Western Europe in 1990 and 2017, and corresponding percent changes.

Cancer site	Age-standardized incidence rate per 100 000 person-years (95% UI)		Age-standardized death rate per 100 000 person-years (95% UI)		Age-standardized DALY rate per 100 000 person-years (95% UI)		Median change (%) between 1990 and 2017		
	1990	2017	1990	2017	1990	2017	Age-standardized incidence rate (95% UI)	**Age-standardized death rate** **(95% UI)**	**Age-standardized DALY rate** **(95% UI)**
Western Europe^b^	357.4 (343.1 – 376.5)	407.7 (381.4 – 441.8)	166.0 (163.6 – 167.1)	128.8 (125.6 – 132.1)	3870.1 (3815.6 – 3919.3)	2877.7 (2790.0 – 2976.9)	13.7 (9.5 – 18.1)	− 22.4 (− 24.3 to − 20.5)	− 25.6 (− 27.6 to − 23.7)
France	350.8 (340.0 – 359.1)	396.4 (351.7 – 450.3)	175.1 (170.8 – 177.2)	131.9 (125.2 – 139.0)	4106.9 (4023.9 – 4169.1)	3018.2 (2856.8 – 3196.9)	12.5 (− 0.1 – 28.1)	− 24.7 (− 28.4 to − 20.7)	− 26.5 (− 30.3 to − 22.3)
Germany	327.5 (319.3 – 333.4)	413.3 (381.3 – 448.5)	166.8 (164.2 – 168.7)	133.6 (121.5 – 146.0)	3931.8 (3871.8 – 3990.2)	3026.5 (2746.6 – 3334.6)	25.1 (15.1 – 35.8)	− 19.9 (− 27.0 to − 12.3)	− 23.0 (− 30.1 to − 15.7)
Italy	341.8 (335.5 – 347.9)	375.5 (355.0 – 397.3)	168.9 (166.8 – 170.6)	121.2 (114.6 – 127.4)	3964.2 (3907.9 – 4020.9)	2676.6 (2519.1 – 2835.7)	9.4 (3.3 – 15.8)	− 28.2 (− 32.2 to − 24.6)	− 32.5 (− 36.4 to − 28.7)
Spain	316.4 (311.3 – 320.8)	347.4 (322.1 – 379.0)	146.5 (144.1 – 148.2)	117.8 (111.5 – 123.4)	3513.5 (3457.3 – 3567.6)	2676.3 (2526.4 – 2823.2)	9.4 (1.4 – 19.5)	− 19.6 (− 23.6 to − 15.8)	− 23.8 (− 28.0 to − 20.0)
United Kingdom	449.2 (401.8 – 510.0)	513.9 (442.1 – 605.3)	179.0 (177.0 – 180.5)	139.3 (137.4 – 141.2)	4088.3 (4021.3 – 4145.3)	3023.2 (2950.4 – 3104.4)	14.6 (9.8 – 19.4)	− 22.1 (− 23.0 to − 21.2)	− 26.1 (− 27.2 to − 24.6)

UI: uncertainty intervals.

^a^Includes malignant neoplasms (International Classification of Diseases 10 [ICD-10] codes C00-C96, excluding Kaposi sarcoma, ICD-10 C46), benign/in situ neoplasms (ICD-10 D00-D49), and other malignant neoplasms (ICD-10 codes C17, C30-C31, C37, C38, C40-C41, C47-C49, C4A, C51-C52, C57-C58, C60, C63, C66, C68, C69, C74-C75). ^b^Includes Andorra, Austria, Belgium, Cyprus, Denmark, Finland, France, Germany, Greece, Iceland, Ireland, Israel, Italy, Luxembourg, Malta, Netherlands, Norway, Portugal, Spain, Sweden, Switzerland, United Kingdom.

## Discussion

This analysis of GBD cancer burden in Italy indicates that over the last decades cancer cases and incidence rates significantly increased overall and for almost all cancer sites, the main exception being stomach cancer. More favorable trends were, however, observed in ASIRs for TBL and as other tobacco-related cancers. Cancer deaths and death rates also increased, but death rates were more favorable—at least over more recent calendar years—for various neoplasms, including stomach, upper aerodigestive tract, Hodgkin lymphoma, and testicular cancer. Moreover, ASDRs steadily declined, particularly in men, for most cancer sites, the main exception being pancreatic cancer. A slight decline in DALYs and DALY rates was observed overall and for several cancer sites, and age-standardized DALY rates significantly declined for most neoplasms. Similar declines were found for YLLs, which explain about 95% of DALYs. Conversely, YLDs and YLD rates significantly increased overall and for many cancer sites, although a decrease in age-standardized YLD rates was observed for some neoplasms, including bladder, stomach, and larynx.

A few considerations may help interpret the patterns of cancer in Italy. For all neoplasms and most cancers considered, we observed an increase in the absolute number of cancer cases and cancer deaths, reflected also in an increase of crude incidence and death rates. Conversely, ASIRs, and particularly ASDRs, showed favorable patterns for most neoplasms considered. The differences between crude and age-standardized rates indicate that the increase in cancer burden in Italy is mainly due to the changes in the population structure over the last decades^1,9^. The decrease in overall YLLs from 1990 to 2017, while the total deaths increased, also indicates that people are dying at older ages. Moreover, the increase in the overall YLDs, along with the decrease in YLLs, are likely due to better survival rates. The decreases in the age-standardized (death) rates for many cancers observed in Italy over the last few decades, can be likely attributable to improvements in modifiable risk factors, implementation of secondary prevention programs, as well as progresses in the management and treatment for various neoplasms. With reference to primary prevention, an important role in the reduction of cancer mortality and DALYs can be due to favorable modifications in behavioral risk factors, including particularly a decrease in (male) tobacco smoking^10–12^ (the major determinant of lung and other tobacco-related neoplasms^11^), a reduction in alcohol consumption (strongly associated to liver and head and neck cancer, and, at a lower extent, to colorectal and breast cancer^13^), and a more affluent and varied diet (an important determinant of colorectal and other digestive tract cancers)^14,15^. The unfavorable trends in pancreatic cancer age-standardized rates may be due to the patterns in overweight/obesity^16^ and diabetes^17^—two known risk factors for this neoplasm—though the prevalence of overweight/obesity in Italy has not steady increased in Italy as in the USA and many other European countries^18,19^. Improved diagnosis may have also had some role, too.

The prevention and treatment of infections, such as Helicobacter pylori (which causes about 78% of stomach cancers), Hepatitis B and C virus (which accounts for most liver cancers), and Human Papilloma Virus (HPV; causally implicated in most cervical cancers) are also likely to have played a relevant role in the cancer mortality reductions^20–24^. Similarly, the improvement of working conditions and the reduction to occupational exposure to various carcinogens (such as asbestos) over the last decades may help explain the reduction in lung and bladder cancer ASDR^25,26^. Indoor and outdoor air pollution, a known risk factor for lung but also bladder cancer, with a synergistic effect with tobacco smoking^27^, has also substantially decreased in Italy over the last few decades, this further contributing to the favorable patterns in those neoplasms.

Screening has been the main responsible of the long-term decline in cervical cancer rates (through the Pap smear test and, more recently, the HPV test) in Italy as in most other Western European countries^28–30^. The implementation of organized screening programs (through fecal occult blood test, flexible sigmoidoscopy, and colonoscopy) has been shown to improve early diagnosis and reduce mortality from colorectal cancer^31,32^. Breast cancer screening (through breast examination and mammography) is also active in Italy since the early 2000s for women over age 50, although the coverage of such screening programs is still low in many Italian regions and its effectiveness is still debated^33,34^. The use of the prostate-specific antigen (PSA) test may have improved early diagnosis of prostate cancer, although the quantification of the role of the PSA test on mortality from this neoplasm is still under debate^35,36^.

A relevant role in the reduction of mortality rates from several cancers (including, among others, breast, prostate, testicular, leukemia, and Hodgkin lymphoma) is also due to the progress in the management and treatment for those cancers over the last decades, with the adoption of modern diagnostic techniques, improvements in surgery, introduction of innovative treatment approaches, recent developments in personalized medicine, as well as a better case management^36–40^.

We observed a relatively lower burden of cancer in Italy, as compared to other most Western European countries, this suggesting a possible improved control of modifiable risk factors^41^, as well as a better management and treatment for cancer in our country. The access to the best treatment options for cancer care in Italy is guaranteed by the presence of an efficient National Health System (NHS) with universal coverage, which assures since 1978 free of charge access to health care for all citizens^42^. Indeed, the Italian NHS ranked second globally by the World Health Organization in 2010, first by the Bloomberg Global Health Index in 2013, and ninth by the GBD Healthcare Access and Quality Index 2016^9,43,44^.

### Limitations

As in prior GBD studies, estimates presented in this study depend on the quality and quantity of the data sources available to inform the estimates^45^. Because of the lag time for data reporting, estimates for 2017 were mainly based on data and trends from recent years. Cancer mortality estimates are predominantly based on vital registration data, cancer registry data, and to a much lesser extent other data sources. In Italy, vital registration data are available since long time, has a 99% coverage, and cancer certification is reasonably reliable and valid, particularly for most common neoplasms^7^. Italian cancer registries cover approximatively 70% of the Italian population^46^. Incidence estimates used in this paper were predicted values from models that used observed mortality data as inputs and the use of MIRs. Although MIRs can change (for example as a consequence of COVID-19), incidence from mortality is estimated after careful estimation of MIRs using selected data sources^1^. Moreover, MIRs allow for a uniform method to estimate incidence and have been used in other cancer estimation frameworks which have detailed its benefits, including greater representativeness, especially in the absence of quality or complete population-based cancer registry systems^47^. Although the proportion of miscoded deaths in Italy is low, the redistribution of unspecified codes (the so-called “garbage” codes) may have somehow affected mortality, as well as incidence, estimates. Misclassification of metastatic sites as primary cancer sites (e.g., liver, TBL, and brain) is another source of potential bias, but again it should be limited in Italy, where sufficient diagnostic resources exist. Further, changes in coding practices or coding systems may also have an effect, even though mapping to the GBD causes list includes adjustments to account for different coding systems. GBD incidence and mortality estimates were somewhat higher than those provided by the Global Cancer Observatory, but differences were below 10% for many cancers, except for selected cancer sites (such as corpus uteri, brain, and stomach for incidence, and nasopharynx, cervix, and prostate for mortality)^6^. Such differences may be due to different estimation methods used and, in particular, to the redistribution of “garbage codes” made by GBD.

## Conclusions

Age-standardized cancer rates in Italy showed favorable patterns over the last few decades, particularly for mortality, thanks to improvements in lifestyles, early diagnosis and treatment. However, the absolute number of cancer cases and, to a lower extent, of cancer deaths increased, likely due to the progressive ageing of the population^1^. Such increase may threaten the already progressively reducing country health resources^48^. Therefore, health care professionals, researchers, and policy makers should made a continuous effort in health promotion and prevention, to maximize the control of behavioral and environmental risk factors (namely tobacco, alcohol, low physical activity, high body mass index, and air pollution) for cancer, but also of other non-communicable diseases, as well as to promote early diagnosis, and further improve cancer treatment and management.

## Methods

Data were extracted from the GBD 2017 results tool (http://ghdx.healthdata.org/gbd-results-tool). Methods and data sources are described in detail in previous GBD publications^1,2,49,50^ and are compliant with the Guidelines for Accurate and Transparent Health Estimates Reporting. Briefly, GBD 2017 comprehensively and systematically analyzed 282 causes of death, 359 diseases and injuries, and 84 behavioral and environmental risks for 195 countries and territories. Here, we focused specifically on the Italian cancer burden and present estimates of incidence, mortality, YLLs, YLDs, and DALYs in Italy for 30 cancer categories (corresponding to GBD Level 3, including malignant neoplasms, International Classification of Diseases 10 [ICD-10] codes C00-C96, excluding Kaposi sarcoma, ICD-10 C46, benign/in situ neoplasms, ICD-10 D00-D49, and other malignant neoplasms, ICD-10 codes C17, C30-C31, C37, C38, C40-C41, C47-C49, C4A, C51-C52, C57-C58, C60, C63, C66, C68, C69, C74-C75)^1^ in the years 1990 and 2017. For a comparative purpose, incidence, morality, and DALYs are also presented for all Western European countries combined, as defined by GBD (i.e., Andorra, Austria, Belgium, Cyprus, Denmark, Finland, France, Germany, Greece, Iceland, Ireland, Israel, Italy, Luxembourg, Malta, Netherlands, Norway, Portugal, Spain, Sweden, Switzerland, United Kingdom), and for the most populated countries of Western Europe (i.e., France, Germany, Spain, and the United Kingdom). This analysis allowed us to compare the Italian cancer burden, with that of other European areas with similar socio-economic conditions.

### Estimation framework

Details of the GBD estimation framework are provided in the eAppendix of the GBD 2017 Cancer Collaboration paper^1^. Briefly, the GBD cancer estimation process starts with mortality. Mortality estimates are based on vital registration data using an ensemble model approach^2,51^. Single-cause mortality estimates are scaled into the separately estimated all-cause estimate^2^. Cancer incidence estimates are derived from mortality estimates, dividing them by a mortality to incidence ratio (MIR). MIR is separately estimated using a spatio-temporal Gaussian process regression for each cancer type, sex, 5-year age group, location, and year^52^. The correlation between survival data and the MIR is used to estimate 10-year cancer prevalence; total prevalence is then partitioned into four sequelae (i.e., diagnosis/treatment, remission, metastatic/disseminated, and terminal phase), and each sequela prevalence is multiplied by a disability weight to estimate YLDs. Lifetime prevalence of procedure-related disability is estimated for larynx, breast, colorectal, bladder, and prostate cancer. YLLs are calculated as the difference between the corresponding standard life expectancy for a person’s age, sex, and year of actual age at death^2^. DALYs are the sum of YLDs and YLLs and represent the loss in years due to premature death or morbidity (one DALY can be regarded as one lost year of “fully healthy life”).

The GBD world population standard is used for the calculation of age-standardized rates^2^. All rates are reported per 100,000 person-years. We also provide 95% uncertainty intervals (UI) for all estimates.

## Supplementary information

Supplementary Information 1.

## Data Availability

Data are available in the GBD 2017 results tool (http://ghdx.healthdata.org/gbd-results-tool).
