# Supplementary material for: National burden of cancer in Italy, 1990–2017: a systematic analysis for the global burden of disease study 2017
Source: Sci Rep. 2020 Dec 16;10:22099. doi: 10.1038/s41598-020-79176-3 (PMC7744506; doi:10.1038/s41598-020-79176-3)
Supplement: Supplementary file 1 — Supplementary Information 1. [file 41598_2020_79176_MOESM1_ESM.docx]

**SUPPLMENTARY MATERIAL**

**NATIONAL BURDEN OF CANCER IN ITALY, 1990-2017: A SYSTEMATIC ANALYSIS FOR THE GLOBAL BURDEN OF DISEASE STUDY 2017**

Cristina Bosetti, Eugenio Traini, Tahiya Alam, Christine A. Allen, Giulia Carreras, Kelly Compton, Christina Fitzmaurice, Lisa M. Force, Silvano Gallus, Giuseppe Gorini, James D. Harvey, Jonathan M. Kocarnik, Carlo La Vecchia, Alessandra Lugo, Mohsen Naghavi, Alyssa Pennini, Cristiano Piccinelli, Luca Ronfani, Rixing Xu, Lorenzo Monasta

**Supplementary Table S1.** Incidence, incidence rates, and age-standardized incidence rates for all cancers and 30 cancer groups, Italy, 2017, and percent change between 1990 and 2017 (ordered by ranking in incident cases in 2017).

| **Cancer site** | **Rank by incident cases in 2017** | | **Incidence**  **(Thousands) (95% UI)** | **Incidence rate per 100 000 person-years (95% UI)** | **Age-standardized incidence rate per 100 000 person-years**  **(95% UI)** | **Median change (%)**  **between 1990 and 2017** | | |
| --- | --- | --- | --- | --- | --- | --- | --- | --- |
|  | **1990** | **2017** | **2017** | **2017** | **2017** | **Incidence**  **(95% UI)** | **Incidence rate**  **(95% UI)** | **Age-standardized incidence rate**  **(95% UI)** |
| All cancers^a^ |  |  | 469.3  (444.6 – 497.5) | 774.5  (733.8 – 821.0) | 375.5  (355.0 – 397.3) | 60.5  (51.6 – 69.2) | 50.4  (42.1 – 58.6) | 9.4  (3.3 – 15.8) |
| Other neoplasm^b^ | 1 | 1 | 197.8  (191.3 – 204.4) | 326.5  (315.7 – 337.4) | 216.1  (209.5 – 222.4) | 27.3  (24.5 – 30.6) | 19.3  (16.7 – 22.4) | 0.0  (-2.0 – 2.2) |
| Non-melanoma skin cancer | 2 | 2 | 74.1  (71.5 – 77.0) | 122.3  (117.9 – 127.0) | 56.1  (54.1 – 58.2) | 96.2  (89.8 – 101.5) | 83.9  (77.9 – 88.9) | 31.8  (27.6 – 35.1) |
| Colon and rectum cancer | 5 | 3 | 52.2  (48.4 – 56.8) | 86.2  (79.9 – 93.8) | 37.2  (34.3 – 40.4) | 69.9  (55.6 – 84.9) | 59.2  (45.9 – 73.3) | 8.8  (-0.5 – 18.7) |
| Breast cancer | 3 | 4 | 50.1  (44.7 – 55.6) | 82.7  (73.8 – 91.7) | 44.2  (39.5 – 49.3) | 39.7  (22.4 – 57.7) | 30.9  (14.8 – 47.8) | -1.6  (-13.8 – 11.1) |
| Tracheal, bronchus, and lung cancer | 4 | 5 | 41.0  (37.2 – 45.2) | 67.6  (61.5 – 74.6) | 29.5  (27.0 – 32.5) | 28.4  (16.2 – 42.9) | 20.4  (8.9 – 33.9) | -16.2  (-24.1 – -7.3) |
| Prostate cancer | 8 | 6 | 40.9  (33.0 – 65.6) | 67.5  (54.5 – 108.2) | 29.4  (23.3 – 47.9) | 133.9  (87.8 – 223.5) | 119.2  (76.0 – 203.2) | 59.2  (26.9 – 122.2) |
| Bladder cancer | 7 | 7 | 23.4  (21.3 – 25.7) | 38.7  (35.2 – 42.4) | 15.9  (14.4 – 17.5) | 29.6  (18.0 – 42.2) | 21.4  (10.6 – 33.3) | -19.4  (-26.8 – -11.5) |
| Stomach cancer | 6 | 8 | 18.6  (16.5 – 21.0) | 30.8  (27.3 – 34.6) | 12.7  (11.3 – 14.3) | -2.6  (-15.0 – 10.5) | -8.7  (-20.3 – 3.6) | -39.3  (-46.5 – -31.6) |
| Leukemia | 9 | 9 | 16.2  (14.7 – 17.6) | 26.7  (24.3 – 29.0) | 14.1  (12.8 – 15.5) | 44.1  (15.0 – 75.5) | 35.1  (7.8 – 64.5) | -14.8  (-30.0 – 1.6) |
| Non-Hodgkin lymphoma | 12 | 10 | 15.0  (13.5 – 16.6) | 24.8  (22.2 – 27.4) | 13.5  (12.1 – 14.9) | 105.0  (80.2 – 130.8) | 92.2  (68.9 – 116.3) | 42.9  (25.7 – 61.4) |
| Other malignant neoplasms^c^ | 11 | 11 | 15.0  (12.3 – 17.2) | 24.7  (20.3 – 28.3) | 14.7  (12.1 – 17.2) | 103.6  (62.9 – 165.2) | 90.8  (52.7 – 148.6) | 47.3  (17.5 – 96.7) |
| Uterine cancer | 22 | 12 | 14.7  (12.7 – 17.0) | 24.2  (20.9 – 28.1) | 12.2  (10.5 – 14.3) | 332.5  (267.9 – 412.4) | 305.4  (244.8 – 380.3) | 207.6  (160.2 – 266.9) |
| Malignant skin melanoma | 15 | 13 | 13.5  (8.3 – 15.9) | 22.3  (13.7 – 26.2) | 14.0  (8.5 – 16.5) | 138.4  (78.7 – 177.7) | 123.5  (67.5 – 160.3) | 83.3  (36.9 – 113.2) |
| Pancreatic cancer | 13 | 14 | 13.5  (12.2 – 14.9) | 22.2  (20.1 – 24.6) | 9.1  (8.3 – 9.9) | 86.2  (65.8 – 109.2) | 74.6  (55.4 – 96.1) | 14.7  (3.0 – 26.9) |
| Liver cancer | 10 | 15 | 12.5  (11.0 – 14.2) | 20.7  (18.2 – 23.4) | 9.1  (8.0 – 10.4) | 47.1  (30.5 – 65.7) | 37.9  (22.3 – 55.3) | -1.8  (-13.0 – 11.0) |
| Kidney cancer | 14 | 16 | 10.7  (9.2 – 12.1) | 17.7  (15.3 – 19.9) | 8.9  (7.6 – 10.0) | 54.5  (35.0 – 75.7) | 44.9  (26.5 – 64.6) | 3.6  (-10.1 – 18.8) |
| Brain and nervous system cancer | 18 | 17 | 9.1  (6.6 – 11.2) | 15.0  (10.9 – 18.5) | 10.0  (7.8 – 12.3) | 87.8  (38.7 – 143.1) | 76.1  (30.0 – 127.8) | 44.2  (9.9 – 90.0) |
| Thyroid cancer | 16 | 18 | 6.7  (5.8 – 7.6) | 11.1  (9.6 – 12.6) | 6.8  (5.9 – 7.8) | 24.8  (5.1 – 45.3) | 17.0  (-1.5 – 36.2) | -4.7  (-19.9 – 11.7) |
| Multiple myeloma | 23 | 19 | 6.0  (5.1 – 7.5) | 9.9  (8.4 – 12.3) | 4.4  (3.7 – 5.5) | 103.1  (66.0 – 136.5) | 90.4  (55.6 – 121.7) | 33.1  (10.6 – 54.8) |
| Gallbladder and biliary tract cancer | 21 | 20 | 5.7  (4.7 – 7.3) | 9.5  (7.8 – 12.1) | 3.7  (3.1 – 4.6) | 42.1  (10.5 – 84.4) | 33.2  (3.6 – 72.8) | -14.8  (-32.7 – 6.5) |
| Ovarian cancer | 20 | 21 | 5.4  (4.8 – 6.1) | 8.9  (7.9 – 10.1) | 4.8  (4.3 – 5.4) | 20.9  (6.8 – 37.7) | 13.3  (0.1 – 29.1) | -13.0  (-23.9 – -0.1) |
| Lip and oral cavity cancer | 19 | 22 | 4.9  (4.4 – 5.4) | 8.0  (7.3 – 8.9) | 3.8  (3.4 – 4.2) | 6.7  (-6.4 – 19.9) | 0.0  (-12.3 – 12.4) | -29.5  (-38.0 – -20.7) |
| Larynx cancer | 17 | 23 | 4.5  (4.0 – 5.1) | 7.4  (6.6 – 8.4) | 3.6  (3.2 – 4.1) | -16.0  (-27.2 – -3.4) | -21.3  (-31.8 – -9.5) | -40.3  (-48.2 – -31.1) |
| Cervical cancer | 25 | 24 | 3.1  (2.7 – 3.6) | 5.1  (4.5 – 5.9) | 3.3  (2.8 – 3.9) | 34.9  (13.9 – 59.2) | 26.5  (6.8 – 49.2) | 5.0  (-13.2 – 27.0) |
| Hodgkin lymphoma | 26 | 25 | 2.6  (2.1 – 3.5) | 4.3  (3.5 – 5.8) | 4.2  (3.3 – 5.7) | 23.1  (-4.1 – 74.4) | 15.4  (-10.1 – 63.4) | 26.6  (-6.2 – 82.2) |
| Esophageal cancer | 24 | 26 | 2.5  (2.2 – 2.7) | 4.1  (3.7 – 4.5) | 1.8  (1.7 – 2.0) | 1.2  (-8.6 – 13.2) | -5.1  (-14.3 – 6.1) | -33.7  (-40.4 – -25.8) |
| Testicular cancer | 28 | 27 | 2.4  (1.9 – 2.9) | 4.0  (3.2 – 4.8) | 4.3  (3.4 – 5.4) | 51.1  (12.3 – 99.9) | 41.7  (5.3 – 87.4) | 62.8  (18.2 – 120.2) |
| Other pharynx cancer | 27 | 28 | 2.1  (1.9 – 2.4) | 3.5  (3.1 – 3.9) | 1.9  (1.7 – 2.1) | 24.1  (10.2 – 39.3) | 16.3  (3.2 – 30.6) | -8.8  (-19.4 – 3.0) |
| Mesothelioma | 29 | 29 | 1.7  (1.6 – 2.0) | 2.9  (2.6 – 3.2) | 1.3  (1.1 – 1.4) | 23.9  (-4.8 – 83.4) | 16.2  (-10.8 – 71.9) | -18.5  (-37.8 – 21.6) |
| Nasopharynx cancer | 30 | 30 | 1.1  (0.9 – 1.3) | 1.8  (1.5 – 2.2) | 1.0  (0.8 – 1.2) | 11.6  (-13.0 – 43.1) | 4.6  (-18.5 – 34.1) | -21.1  (-38.8 – 2.2) |

UI: uncertainty intervals.

^a^Includes malignant neoplasms (International Classification of Diseases 10 [ICD-10] codes C00-C96, excluding Kaposi sarcoma, ICD-10 C46), benign/in situ neoplasms (ICD-10 D00-D49), and other malignant neoplasms (ICD-10 codes C17, C30-C31, C37, C38, C40-C41, C47-C49, C4A, C51-C52, C57-C58, C60, C63, C66, C68, C69, C74-C75). ^b^Includes benign/in situ neoplasms (ICD-10 D00-D49). ^c^Includes other malignant neoplasms (ICD-10 codes C17, C30-C31, C37, C38, C40-C41, C47-C49, C4A, C51-C52, C57-C58, C60, C63, C66, C68, C69, C74-C75).

**Supplementary Table S2.** Number of deaths, death rates, and age-standardized death rates for all cancers and 30 cancer groups, Italy, 2017, and percent change between 1990 and 2017 (ordered by ranking in number of deaths in 2017).

| **Cancer site** | **Rank by number of deaths in 2017** | | **Deaths**  **(Thousands) (95% UI)** | **Death rate per**  **100 000 person-years**  **(95% UI)** | **Age-standardized death rate per**  **100 000 person-years**  **(95% UI)** | **Median change (%)**  **between 1990 and 2017** | | |
| --- | --- | --- | --- | --- | --- | --- | --- | --- |
|  | **1990** | **2017** | **2017** | **2017** | **2017** | **Deaths**  **(95% UI)** | **Death rate**  **(95% UI)** | **Age-standardized death rate**  **(95% UI)** |
| All cancers^a^ |  |  | 180.6  (171.0 – 189.9) | 298.0  (282.1 – 313.4) | 121.2  (114.6 – 127.4) | 18.5  (12.2 – 24.5) | 11.1  (5.1 – 16.7) | -28.2  (-32.2 – -24.6) |
| Tracheal, bronchus, and lung cancer | 1 | 1 | 34.1  (31.9 – 36.3) | 56.3  (52.7 – 60.0) | 23.5  (22.0 – 25.1) | 6.3  (-0.8 – 13.8) | -0.3  (-7.0 – 6.7) | -32.8  (-37.4 – -28.0) |
| Colon and rectum cancer | 3 | 2 | 21.0  (19.5 – 22.6) | 34.6  (32.2 – 37.3) | 13.3  (12.3 – 14.3) | 30.1  (20.3 – 40.1) | 21.9  (12.8 – 31.3) | -24.2  (-29.8 – -18.2) |
| Breast cancer | 4 | 3 | 13.0  (11.8 – 14.2) | 21.5  (19.5 – 23.4) | 9.2  (8.3 – 10.0) | 11.2  (0.7 – 21.1) | 4.2  (-5.6 – 13.5) | -32.5  (-39.0 – -26.3) |
| Pancreatic cancer | 6 | 4 | 12.7  (11.8 – 13.7) | 20.9  (19.4 – 22.6) | 8.4  (7.8 – 9.1) | 65.6  (53.1 – 79.1) | 55.2  (43.5 – 67.9) | 1.2  (-6.7 – 9.6) |
| Stomach cancer | 2 | 5 | 12.1  (11.3 – 13.0) | 20.0  (18.6 – 21.4) | 7.7  (7.2 – 8.3) | -32.1  (-37.2 – -27.1) | -36.4  (-41.1 – -31.7) | -60.0  (-62.9 – -57.1) |
| Liver cancer | 5 | 6 | 10.6  (9.6 – 11.7) | 17.5  (15.9 – 19.3) | 7.1  (6.4 – 7.9) | 17.3  (6.9 – 28.9) | 9.9  (0.2 – 20.8) | -26.9  (-33.6 – -19.5) |
| Prostate cancer | 7 | 7 | 9.7  (8.2 – 14.2) | 16.0  (13.5 – 23.5) | 5.4  (4.6 – 8.2) | 55.7  (36.6 – 97.3) | 46.0  (28.0 – 84.9) | -16.7  (-27.5 – 8.5) |
| Bladder cancer | 8 | 8 | 7.6  (7.0 – 8.4) | 12.6  (11.5 – 13.8) | 4.4  (4.0 – 4.8) | 27.6  (16.6 – 39.8) | 19.6  (9.3 – 31.1) | -29.9  (-35.8 – -23.3) |
| Leukemia | 9 | 9 | 7.6  (7.1 – 8.2) | 12.6  (11.7 – 13.5) | 5.4  (5.0 – 5.8) | 31.0  (20.2 – 41.2) | 22.8  (12.7 – 32.3) | -23.4  (-29.6 – -17.7) |
| Non-Hodgkin lymphoma | 11 | 10 | 5.4  (5.0 – 5.8) | 8.9  (8.2 – 9.6) | 3.7  (3.4 – 4.0) | 42.9  (31.0 – 54.7) | 33.9  (22.8 – 45.0) | -14.2  (-21.5 – -7.0) |
| Other malignant neoplasms^b^ | 10 | 11 | 5.3  (4.4 – 5.8) | 8.8  (7.3 – 9.6) | 4.0  (3.3 – 4.4) | 8.8  (-1.5 – 34.9) | 2.0  (-7.7 – 26.4) | -31.4  (-38.7 – -12.1) |
| Other neoplasms^c^ | 19 | 12 | 4.7  (3.1 – 6.4) | 7.8  (5.1 – 10.5) | 3.0  (2.0 – 4.2) | 129.7  (50.7 – 199.1) | 115.3  (41.2 – 180.3) | 29.0  (-14.8 – 61.4) |
| Kidney cancer | 15 | 13 | 4.3  (3.7 – 4.8) | 7.1  (6.0 – 7.8) | 2.9  (2.5 – 3.2) | 63.8  (45.4 – 79.6) | 53.5  (36.3 – 68.3) | -1.4  (-11.2 – 8.1) |
| Gallbladder and biliary tract cancer | 12 | 14 | 4.3  (3.8 – 4.7) | 7.1  (6.2 – 7.8) | 2.7  (2.3 – 2.9) | 22.9  (6.8 – 33.3) | 15.2  (0.1 – 24.9) | -28.4  (-39.0 – -22.2) |
| Brain and nervous system cancer | 13 | 15 | 4.0  (2.9 – 4.5) | 6.7  (4.7 – 7.5) | 3.6  (2.7 – 4.0) | 25.3  (-9.9 – 40.4) | 17.4  (-15.6 – 31.6) | -12.6  (-33.5 – 4.1) |
| Ovarian cancer | 14 | 16 | 3.8  (3.4 – 4.3) | 6.3  (5.7 – 7.0) | 2.8  (2.5 – 3.1) | 27.3  (13.4 – 42.0) | 19.3  (6.3 – 33.1) | -18.7  (-27.8 – -8.7) |
| Multiple myeloma | 18 | 17 | 3.6  (3.2 – 4.4) | 6.0  (5.3 – 7.3) | 2.3  (2.0 – 2.9) | 69.6  (43.2 – 90.2) | 59.0  (34.2 – 78.2) | 1.7  (-13.1 – 13.4) |
| Esophageal cancer | 17 | 18 | 2.2  (2.0 – 2.3) | 3.6  (3.3 – 3.9) | 1.5  (1.4 – 1.6) | -13.6  (-21.2 – -6.1) | -19.0  (-26.1 – -12.0) | -46.1  (-50.8 – -41.3) |
| Malignant skin melanoma | 21 | 19 | 2.1  (1.3 – 2.4) | 3.5  (2.1 – 4.0) | 1.6  (1.0 – 1.8) | 55.2  (14.1 – 75.5) | 45.5  (7.0 – 64.5) | -0.9  (-26.2 – 12.0) |
| Lip and oral cavity cancer | 20 | 20 | 1.9  (1.7 – 2.0) | 3.1  (2.9 – 3.3) | 1.3  (1.2 – 1.4) | 0.4  (-7.9 – 8.9) | -5.9  (-13.7 – 2.0) | -38.3  (-43.5 – -32.9) |
| Mesothelioma | 22 | 21 | 1.7  (1.6 – 1.9) | 2.8  (2.6 – 3.1) | 1.2  (1.1 – 1.3) | 36.8  (9.3 – 97.3) | 28.2  (2.4 – 84.9) | -13.9  (-31.0 – 23.9) |
| Cervical cancer | 23 | 22 | 1.6  (1.5 – 1.8) | 2.7  (2.4 – 3.0) | 1.2  (1.1 – 1.3) | 45.0  (29.2 – 61.6) | 35.9  (21.1 – 51.5) | -8.4  (-18.5 – 2.0) |
| Uterine cancer | 28 | 23 | 1.6  (1.4 – 1.8) | 2.6  (2.4 – 3.0) | 1.1  (1.0 – 1.2) | 177.1  (146.4 – 212.9) | 159.7  (131.0 – 193.3) | 71.4  (51.4 – 95.1) |
| Larynx cancer | 16 | 24 | 1.6  (1.5 – 1.7) | 2.6  (2.4 – 2.9) | 1.1  (1.0 – 1.2) | -36.7  (-42.3 – -30.7) | -40.7  (-45.9 – -35.1) | -59.6  (-63.3 – -55.7) |
| Non-melanoma skin cancer | 25 | 25 | 1.2  (1.1 – 1.3) | 2.0  (1.8 – 2.1) | 0.7  (0.6 – 0.7) | 81.3  (67.6 – 97.2) | 70.0  (57.1 – 84.8) | -9.6  (-16.8 – -1.3) |
| Other pharynx cancer | 24 | 26 | 0.9  (0.8 – 1.0) | 1.4  (1.3 – 1.6) | 0.7  (0.6 – 0.8) | -3.0  (-12.1 – 7.4) | -9.1  (-17.6 – 0.7) | -32.3  (-38.9 – -24.5) |
| Nasopharynx cancer | 29 | 27 | 0.6  (0.6 – 0.7) | 1.1  (1.0 – 1.2) | 0.5  (0.4 – 0.5) | 20.8  (8.9 – 34.8) | 13.2  (2.1 – 26.3) | -22.5  (-30.2 – -13.4) |
| Thyroid cancer | 27 | 28 | 0.6  (0.6 – 0.7) | 1.0  (0.9 – 1.1) | 0.4  (0.4 – 0.5) | 6.9  (-3.6 – 16.8) | 0.2  (-9.7 – 9.5) | -35.1  (-41.5 – -28.8) |
| Hodgkin lymphoma | 26 | 29 | 0.4  (0.4 – 0.6) | 0.7  (0.6 – 0.9) | 0.4  (0.3 – 0.5) | -29.6  (-36.5 – -14.5) | -34.0  (-40.5 – -19.8) | -50.2  (-55.8 – -37.1) |
| Testicular cancer | 30 | 30 | 0.1  (0.1 – 0.1) | 0.2  (0.1 – 0.2) | 0.1  (0.1 – 0.1) | -14.6  (-26.7 – -1.2) | -19.9  (-31.3 – -7.4) | -30.4  (-41.3 – -18.5) |

UI: uncertainty intervals.

^a^Includes malignant neoplasms (International Classification of Diseases 10 [ICD-10] codes C00-C96, excluding Kaposi sarcoma, ICD-10 C46), benign/in situ neoplasms (ICD-10 D00-D49), and other malignant neoplasms (ICD-10 codes C17, C30-C31, C37, C38, C40-C41, C47-C49, C4A, C51-C52, C57-C58, C60, C63, C66, C68, C69, C74-C75). ^b^Includes other malignant neoplasms (ICD-10 codes C17, C30-C31, C37, C38, C40-C41, C47-C49, C4A, C51-C52, C57-C58, C60, C63, C66, C68, C69, C74-C75). ^c^Includes benign/in situ neoplasms (ICD-10 D00-D49).

**Supplementary Table S3.** Contribution of years lived with disability (YLDs) and years of life lost (YLLs) to disability-adjusted life-years (DALYs) for all cancers and 30 cancer groups, Italy, 2017 (ordered by ranking in number of DALYs in 2017).

| **Cancer site** | **DALYs** | **YLDs (%)** | **YLLs (%)** |
| --- | --- | --- | --- |
| All cancers^a^ | 3203988.7 | 192094.8 (6.0) | 3011893.9 (94.0) |
| Tracheal, bronchus, and lung cancer | 612436.2 | 10280.6 (1.7) | 602155.6 (98.3) |
| Colon and rectum cancer | 345223.7 | 26569.6 (7.7) | 318654.1 (92.3) |
| Breast cancer | 276178.4 | 36153.6 (13.1) | 240024.8 (86.9) |
| Pancreatic cancer | 210986.0 | 2567.4 (1.2) | 208418.6 (98.8) |
| Stomach cancer | 190697.2 | 5170.8 (2.7) | 185526.4 (97.3) |
| Liver cancer | 182169.1 | 3014.8 (1.7) | 179154.3 (98.3) |
| Prostate cancer | 140811.2 | 26321.6 (18.7) | 114489.6 (81.3) |
| Leukemia | 137949.4 | 6174.5 (4.5) | 131774.9 (95.5) |
| Other malignant neoplasms^b^ | 111094.6 | 10785.9 (9.7) | 100308.7 (90.3) |
| Bladder cancer | 110752.1 | 12288.7 (11.1) | 98463.4 (88.9) |
| Brain and nervous system cancer | 102615.4 | 4094.6 (4.0) | 98520.8 (96.0) |
| Non-Hodgkin lymphoma | 99382.7 | 6259.7 (6.3) | 93123.0 (93.7) |
| Ovarian cancer | 76287.0 | 3170.7 (4.2) | 73116.3 (95.8) |
| Kidney cancer | 75306.2 | 3726.6 (4.9) | 71579.6 (95.1) |
| Other neoplasms^c^ | 71546.9 | 620.5 (0.9) | 70926.5 (99.1) |
| Gallbladder and biliary tract cancer | 65158.9 | 1316.1 (2.0) | 63842.8 (98.0) |
| Multiple myeloma | 60370.4 | 3826.8 (6.3) | 56543.6 (93.7) |
| Malignant skin melanoma | 49315.9 | 6322.3 (12.8) | 42993.6 (87.2) |
| Esophageal cancer | 39353.1 | 728.8 (1.9) | 38624.3 (98.1) |
| Lip and oral cavity cancer | 36325.9 | 2099.6 (5.8) | 34226.3 (94.2) |
| Uterine cancer | 34639.9 | 7664.6 (22.1) | 26975.2 (77.9) |
| Cervical cancer | 33146.5 | 1525.0 (4.6) | 31621.5 (95.4) |
| Larynx cancer | 31864.5 | 2604.3 (8.2) | 29260.1 (91.8) |
| Mesothelioma | 30174.3 | 736.8 (2.4) | 29437.4 (97.6) |
| Other pharynx cancer | 20948.4 | 764.8 (3.7) | 20183.7 (96.3) |
| Non-melanoma skin cancer | 15084.4 | 549.5 (3.6) | 14534.8 (96.4) |
| Thyroid cancer | 13978.3 | 3503.1 (25.1) | 10475.2 (74.9) |
| Nasopharynx cancer | 13484.1 | 555.9 (4.1) | 12928.2 (95.9) |
| Hodgkin lymphoma | 12393.3 | 1428.0 (11.5) | 10965.3 (88.5) |
| Testicular cancer | 4314.9 | 1269.5 (29.4) | 3045.4 (70.6) |

^a^Includes malignant neoplasms (International Classification of Diseases 10 [ICD-10] codes C00-C96, excluding Kaposi sarcoma, ICD-10 C46), benign/in situ neoplasms (ICD-10 D00-D49), and other malignant neoplasms (ICD-10 codes C17, C30-C31, C37, C38, C40-C41, C47-C49, C4A, C51-C52, C57-C58, C60, C63, C66, C68, C69, C74-C75). ^b^Includes other malignant neoplasms (ICD-10 codes C17, C30-C31, C37, C38, C40-C41, C47-C49, C4A, C51-C52, C57-C58, C60, C63, C66, C68, C69, C74-C75). ^c^Includes benign/in situ neoplasms (ICD-10 D00-D49).

**Supplementary Table S4.** Disability adjusted life years (DALYs), DALY rates, and age-standardized DALY rates for all cancers and 30 cancer groups, Italy, 2017, and percent change between 1990 and 2017 (ordered by ranking in number of DALYs in 2017).

| **Cancer site** | **Rank by number of DALYs in 2017** | | **DALYs**  **(Thousands) (95% UI)** | **DALY rate per**  **100 000 person-years (95% UI)** | **Age-standardized DALY rate per**  **100 000 person-years**  **(95% UI)** | **Median change (%)**  **between 1990 and 2017** | | |
| --- | --- | --- | --- | --- | --- | --- | --- | --- |
|  | **1990** | **2017** | **2017** | **2017** | **2017** | **DALYs**  **(95% UI)** | **DALY rate**  **(95% UI)** | **Age-standardized DALY rate**  **(95% UI)** |
| All cancers^a^ |  |  | 3204.0  (3018.2 – 3395.8) | 5287.3  (4980.7 – 5603.8) | 2676.6  (2519.1 – 2835.7) | -3.4  (-8.9 – 1.8) | -9.4  (-14.6 – -4.6) | -32.5  (-36.4 – -28.7) |
| Tracheal, bronchus, and lung cancer | 1 | 1 | 612.4  (573.3 – 653.8) | 1010.7  (946.1 – 1079.0) | 486.8  (455.9 – 520.1) | -14.9  (-20.7 – -8.8) | -20.2  (-25.7 – -14.6) | -40.9  (-44.9 – -36.6) |
| Colon and rectum cancer | 3 | 2 | 345.2  (319.0 – 376.3) | 569.7  (526.5 – 621.0) | 264.7  (244.4 – 288.7) | 9.7  (1.1 – 18.6) | 2.8  (-5.2 – 11.2) | -25.9  (-31.7 – -19.6) |
| Breast cancer | 4 | 3 | 276.2  (248.3 – 304.5) | 455.8  (409.7 – 502.6) | 244.8  (219.2 – 270.7) | -8.3  (-17.4 – 0.4) | -14.0  (-22.6 – -5.9) | -34.9  (-41.4 – -28.4) |
| Pancreatic cancer | 6 | 4 | 211.0  (195.9 – 229.3) | 348.2  (323.3 – 378.3) | 164.5  (152.5 – 178.9) | 39.7  (28.4 – 51.7) | 31.0  (20.3 – 42.2) | -3.7  (-11.1 – 4.7) |
| Stomach cancer | 2 | 5 | 190.7  (176.4 – 206.0) | 314.7  (291.1 – 340.0) | 147.7  (136.5 – 159.7) | -42.2  (-46.6 – -37.7) | -45.8  (-49.9 – -41.6) | -60.4  (-63.4 – -57.2) |
| Liver cancer | 5 | 6 | 182.2  (164.5 – 202.2) | 300.6  (271.4 – 333.6) | 143.1  (128.9 – 159.3) | -0.6  (-10.2 – 10.1) | -6.8  (-15.8 – 3.2) | -30.5  (-37.3 – -22.9) |
| Prostate cancer | 10 | 7 | 140.8  (118.1 – 217.8) | 232.4  (194.8 – 359.4) | 91.7  (76.2 – 146.1) | 35.2  (16.3 – 78.5) | 26.7  (9.0 – 67.3) | -14.9  (-27.4 – 14.3) |
| Leukemia | 7 | 8 | 137.9  (127.1 – 148.9) | 227.6  (209.7 – 245.6) | 142.4  (131.2 – 154.4) | -8.1  (-15.3 – -0.7) | -13.8  (-20.6 – -6.9) | -35.5  (-40.9 – -29.4) |
| Other malignant neoplasms^b^ | 8 | 9 | 111.1  (92.0 – 126.2) | 183.3  (151.8 – 208.2) | 119.8  (99.5 – 139.1) | -7.5  (-19.1 – 19.2) | -13.3  (-24.2 – 11.7) | -28.6  (-38.7 – -6.7) |
| Bladder cancer | 9 | 10 | 110.8  (100.4 – 122.1) | 182.8  (165.7 – 201.6) | 76.1  (68.7 – 84.1) | -0.9  (-9.5 – 8.7) | -7.1  (-15.2 – 1.9) | -36.9  (-42.4 – -31.1) |
| Brain and nervous system cancer | 11 | 11 | 102.6  (79.8 – 117.8) | 169.3  (131.7 – 194.3) | 118.0  (95.7 – 141.7) | 5.3  (-20.1 – 27.3) | -1.3  (-25.1 – 19.3) | -18.8  (-35.3 – 4.8) |
| Non-Hodgkin lymphoma | 12 | 12 | 99.4  (90.8 – 107.9) | 164.0  (149.9 – 178) | 90.7  (82.4 – 98.8) | 7.4  (-1.9 – 16.9) | 0.7  (-8.0 – 9.6) | -25.2  (-32.3 – -18.6) |
| Ovarian cancer | 13 | 13 | 76.3  (67.9 – 85.5) | 125.9  (112.0 – 141.1) | 66.7  (59.3 – 75.3) | 6.3  (-5.5 – 18.8) | -0.4  (-11.4 – 11.4) | -23.1  (-31.6 – -13.6) |
| Kidney cancer | 16 | 14 | 75.3  (65.6 – 83.2) | 124.3  (108.2 – 137.2) | 62.8  (54.7 – 69.6) | 25.8  (13.0 – 38.5) | 17.9  (5.9 – 29.8) | -13.5  (-22.4 – -4.2) |
| Other neoplasms^c^ | 19 | 15 | 71.5  (48.2 – 102.1) | 118.1  (79.5 – 168.5) | 63.6  (42.3 – 92.2) | 62.6  (14.1 – 97.2) | 52.4  (6.9 – 84.8) | 9.8  (-23.1 – 38.6) |
| Gallbladder and biliary tract cancer | 14 | 16 | 65.2  (56.4 – 71.4) | 107.5  (93.0 – 117.8) | 48.3  (41.7 – 53.0) | -0.2  (-12.9 – 8.7) | -6.5  (-18.3 – 1.9) | -33.5  (-41.9 – -26.8) |
| Multiple myeloma | 20 | 17 | 60.4  (53.1 – 76.1) | 99.6  (87.6 – 125.6) | 45.7  (40.1 – 57.8) | 42.4  (24.2 – 57.8) | 33.5  (16.4 – 47.9) | -3.5  (-15.2 – 6.8) |
| Malignant skin melanoma | 21 | 18 | 49.3  (29.9 – 58.1) | 81.4  (49.3 – 95.9) | 48.8  (29.1 – 57.9) | 32.3  (-0.6 – 50.1) | 24.1  (-6.8 – 40.7) | -0.6  (-24.8 – 13.1) |
| Esophageal cancer | 17 | 19 | 39.4  (36.2 – 43) | 64.9  (59.7 – 71.0) | 32.2  (29.6 – 35.4) | -28  (-34.4 – -21.2) | -32.5  (-38.5 – -26.1) | -49.4  (-54.0 – -44.4) |
| Lip and oral cavity cancer | 18 | 20 | 36.3  (33.6 – 39.5) | 59.9  (55.4 – 65.2) | 31.2  (28.7 – 34.0) | -20.8  (-27.9 – -13.5) | -25.7  (-32.4 – -18.9) | -43.9  (-49.1 – -38.6) |
| Uterine cancer | 28 | 21 | 34.6  (30 – 40) | 57.2  (49.4 – 66.1) | 28.2  (24.3 – 32.7) | 164.7  (129.7 – 202.5) | 148.1  (115.3 – 183.6) | 88.2  (61.7 – 116.3) |
| Cervical cancer | 22 | 22 | 33.1  (29.4 – 37.0) | 54.7  (48.5 – 61.0) | 31.1  (27.5 – 34.8) | 21.3  (7.1 – 35.4) | 13.7  (0.4 – 27) | -10.4  (-21.3 – 1.2) |
| Larynx cancer | 15 | 23 | 31.9  (29.1 – 35.0) | 52.6  (48.0 – 57.7) | 26.2  (23.8 – 28.8) | -47.2  (-52.3 – -41.9) | -50.5  (-55.3 – -45.5) | -62.6  (-66.4 – -58.9) |
| Mesothelioma | 23 | 24 | 30.2  (27.3 – 33.4) | 49.8  (45.1 – 55.1) | 23.4  (21.2 – 25.9) | 11.0  (-11.2 – 62.3) | 4.0  (-16.8 – 52.1) | -23.7  (-39.2 – 11.5) |
| Other pharynx cancer | 24 | 25 | 20.9  (18.9 – 23.2) | 34.6  (31.3 – 38.4) | 19.1  (17.2 – 21.2) | -13.6  (-22.3 – -3.5) | -19.0  (-27.2 – -9.6) | -35.9  (-42.5 – -28.5) |
| Non-melanoma skin cancer | 29 | 26 | 15.1  (13.8 – 16.6) | 24.9  (22.7 – 27.5) | 10.9  (9.9 – 12.2) | 32.4  (21.3 – 45.5) | 24.1  (13.7 – 36.3) | -17.6  (-25.3 – -8.8) |
| Thyroid cancer | 26 | 27 | 14.0  (12.3 – 15.8) | 23.1  (20.2 – 26.1) | 12.1  (10.5 – 14) | -6.8  (-16.2 – 3.6) | -12.6  (-21.4 – -2.9) | -32.9  (-40.2 – -24.5) |
| Nasopharynx cancer | 27 | 28 | 13.5  (12.1 – 15.2) | 22.3  (20.0 – 25) | 12.7  (11.4 – 14.3) | -4.8  (-14.8 – 6.6) | -10.8  (-20.1 – -0.1) | -30.2  (-37.9 – -21.2) |
| Hodgkin lymphoma | 25 | 29 | 12.4  (10.1 – 16.4) | 20.5  (16.7 – 27.0) | 16.2  (13.1 – 21.1) | -41.9  (-49.3 – -24.4) | -45.6  (-52.5 – -29.1) | -48.8  (-56.2 – -32.8) |
| Testicular cancer | 30 | 30 | 4.3  (3.6 – 5.2) | 7.1  (5.9 – 8.6) | 7.1  (5.7 – 8.8) | -17.4  (-32.1 – -0.2) | -22.6  (-36.3 – -6.4) | -15.3  (-31.7 – 4.0) |

UI: uncertainty intervals.

^a^Includes malignant neoplasms (International Classification of Diseases 10 [ICD-10] codes C00-C96, excluding Kaposi sarcoma, ICD-10 C46), benign/in situ neoplasms (ICD-10 D00-D49), and other malignant neoplasms (ICD-10 codes C17, C30-C31, C37, C38, C40-C41, C47-C49, C4A, C51-C52, C57-C58, C60, C63, C66, C68, C69, C74-C75). ^b^Includes other malignant neoplasms (ICD-10 codes C17, C30-C31, C37, C38, C40-C41, C47-C49, C4A, C51-C52, C57-C58, C60, C63, C66, C68, C69, C74-C75). ^c^Includes benign/in situ neoplasms (ICD-10 D00-D49).

**Supplementary Table S5.** Number of years of life lost (YLLs), YLL rates, and age-standardized YLL rates for all cancers and 30 cancer groups, Italy, 2017, and percent change between 1990 and 2017 (ordered by ranking in number of YLLs in 2017).

| **Cancer site** | **Rank by number of YLLs in 2017** | | **YLLs**  **(in Thousands) (95% UI)** | **YLL rate per**  **100 000 person-years (95% UI)** | **Age-standardized YLL rate per**  **100 000 person-years**  **(95% UI)** | **Median change (%)**  **between 1990 and 2017** | | |
| --- | --- | --- | --- | --- | --- | --- | --- | --- |
|  | **1990** | **2017** | **2017** | **2017** | **2017** | **YLLs**  **(95% UI)** | **YLL rate**  **(95% UI)** | **Age-standardized YLL rate**  **(95% UI)** |
| All cancers^a^ |  |  | 3011.9  (2842.3 – 3172.1) | 4970.3  (4690.5 – 5234.6) | 2511.5  (2369.3 – 2653.7) | -5.9  (-11.2 – -0.9) | -11.8  (-16.8 – -7.1) | -34.4  (-38.2 – -30.8) |
| Tracheal, bronchus, and lung cancer | 1 | 1 | 602.2  (564 – 644.8) | 993.7  (930.7 – 1064.1) | 479.1  (449.3 – 511.8) | -15.5  (-21.3 – -9.4) | -20.8  (-26.2 – -15.0) | -41.2  (-45.2 – -37.1) |
| Colon and rectum cancer | 3 | 2 | 318.7  (294.7 – 344.5) | 525.9  (486.3 – 568.6) | 244.9  (226.2 – 265.4) | 6.2  (-1.9 – 14.8) | -0.4  (-8.1 – 7.6) | -28.1  (-33.8 – -22.1) |
| Breast cancer | 4 | 3 | 240.0  (216.9 – 262.4) | 396.1  (357.9 – 433.1) | 212.6  (191.6 – 233.4) | -12.9  (-21.6 – -4.8) | -18.4  (-26.5 – -10.8) | -38.2  (-44.3 – -32.4) |
| Pancreatic cancer | 6 | 4 | 208.4  (193.9 – 226.5) | 343.9  (319.9 – 373.7) | 162.7  (151.0 – 176.9) | 39.3  (28.0 – 51.4) | 30.6  (20.0 – 41.9) | -3.8  (-11.3 – 4.5) |
| Stomach cancer | 2 | 5 | 185.5  (172.0 – 199.6) | 306.2  (283.9 – 329.4) | 143.9  (133.1 – 155.4) | -42.9  (-47.2 – -38.5) | -46.5  (-50.5 – -42.4) | -60.8  (-63.9 – -57.7) |
| Liver cancer | 5 | 6 | 179.2  (161.9 – 199.0) | 295.6  (267.1 – 328.4) | 140.9  (126.9 – 157) | -1.2  (-10.8 – 9.3) | -7.4  (-16.4 – 2.4) | -30.9  (-37.7 – -23.5) |
| Leukemia | 7 | 7 | 131.8  (121.6 – 142.3) | 217.5  (200.7 – 234.8) | 137.0  (126.2 – 148.9) | -9.6  (-16.7 – -2.3) | -15.3  (-22.0 – -8.4) | -36.2  (-41.7 – -30.2) |
| Prostate cancer | 11 | 8 | 114.5  (97.3 – 178.8) | 188.9  (160.6 – 295.1) | 72.4  (61.1 – 115.8) | 23.7  (7.0 – 62.7) | 16.0  (0.3 – 52.5) | -24.2  (-35.0 – 1.2) |
| Other malignant neoplasms^b^ | 8 | 9 | 100.3  (83.1 – 112.6) | 165.5  (137.1 – 185.9) | 107.2  (89.4 – 124.1) | -14.4  (-24.4 – 10.5) | -19.8  (-29.2 – 3.5) | -34.4  (-43.4 – -13.3) |
| Brain and nervous system cancer | 10 | 10 | 98.5  (76.4 – 112.9) | 162.6  (126.1 – 186.3) | 113.1  (92.0 – 135.8) | 3.0  (-21.5 – 24.6) | -3.5  (-26.4 – 16.8) | -20.6  (-36.7 – 2.3) |
| Bladder cancer | 9 | 11 | 98.5  (89.4 – 108.2) | 162.5  (147.6 – 178.5) | 67.2  (61.0 – 73.8) | -3.6  (-11.8 – 5.6) | -9.6  (-17.4 – -1.1) | -38.9  (-44.2 – -33.3) |
| Non-Hodgkin lymphoma | 12 | 12 | 93.1  (85.3 – 101.1) | 153.7  (140.8 – 166.9) | 84.7  (77.3 – 92.2) | 3.9  (-5.2 – 13.0) | -2.6  (-11.1 – 5.9) | -27.8  (-34.7 – -21.5) |
| Ovarian cancer | 13 | 13 | 73.1  (65.1 – 82) | 120.7  (107.4 – 135.3) | 63.7  (56.7 – 71.7) | 5.7  (-6.0 – 18.6) | -0.9  (-11.9 – 11.2) | -23.7  (-32.1 – -14.3) |
| Kidney cancer | 15 | 14 | 71.6  (62.2 – 78.9) | 118.1  (102.7 – 130.2) | 59.5  (51.5 – 65.9) | 24.7  (12.2 – 37.0) | 16.9  (5.2 – 28.4) | -14.3  (-23 – -5.5) |
| Other neoplasms^c^ | 19 | 15 | 70.9  (47.6 – 101.5) | 117.0  (78.6 – 167.5) | 63.1  (41.9 – 91.7) | 62.6  (13.7 – 97.7) | 52.4  (6.6 – 85.3) | 9.8  (-23.3 – 39.0) |
| Gallbladder and biliary tract cancer | 14 | 16 | 63.8  (55.2 – 70.0) | 105.4  (91.0 – 115.6) | 47.4  (40.8 – 52.0) | -0.9  (-13.2 – 8.1) | -7.1  (-18.7 – 1.3) | -33.8  (-42.0 – -27.4) |
| Multiple myeloma | 20 | 17 | 56.5  (49.4 – 70.9) | 93.3  (81.5 – 117.1) | 42.5  (37.1 – 53.5) | 38.8  (20.6 – 54.0) | 30.1  (13.1 – 44.3) | -6.4  (-17.6 – 3.4) |
| Malignant skin melanoma | 21 | 18 | 43.0  (26.5 – 49.6) | 70.9  (43.7 – 81.8) | 42.0  (25.6 – 48.9) | 23.9  (-6.9 – 40.1) | 16.2  (-12.8 – 31.4) | -7.8  (-30 – 4.7) |
| Esophageal cancer | 17 | 19 | 38.6  (35.5 – 42.1) | 63.7  (58.6 – 69.5) | 31.7  (29.0 – 34.7) | -28.5  (-34.9 – -21.7) | -32.9  (-39 – -26.6) | -49.7  (-54.3 – -44.8) |
| Lip and oral cavity cancer | 18 | 20 | 34.2  (31.7 – 37.1) | 56.5  (52.3 – 61.2) | 29.5  (27.1 – 32.1) | -22.1  (-29.0 – -14.9) | -27.0  (-33.5 – -20.2) | -44.7  (-49.8 – -39.5) |
| Cervical cancer | 23 | 21 | 31.6  (27.9 – 35.2) | 52.2  (46.1 – 58.1) | 29.4  (26 – 33.0) | 20.7  (6.6 – 34.5) | 13.2  (0.0 – 26.1) | -11.3  (-22 – -0.1) |
| Mesothelioma | 22 | 22 | 29.4  (26.7 – 32.6) | 48.6  (44.0 – 53.8) | 22.9  (20.7 – 25.3) | 10.8  (-11.5 – 62.0) | 3.8  (-17.1 – 51.9) | -23.8  (-39.2 – 11.2) |
| Larynx cancer | 16 | 23 | 29.3  (26.6 – 32.0) | 48.3  (43.8 – 52.8) | 24.0  (21.8 – 26.3) | -49.0  (-53.8 – -43.9) | -52.2  (-56.7 – -47.4) | -64.0  (-67.4 – -60.3) |
| Uterine cancer | 28 | 24 | 27.0  (23.9 – 30.6) | 44.5  (39.4 – 50.5) | 21.7  (19.0 – 24.8) | 138.8  (109.7 – 172.4) | 123.8  (96.5 – 155.3) | 68.6  (47.6 – 92.6) |
| Other pharynx cancer | 24 | 25 | 20.2  (18.3 – 22.5) | 33.3  (30.2 – 37.1) | 18.4  (16.5 – 20.4) | -14.7  (-23.4 – -4.7) | -20.1  (-28.2 – -10.7) | -36.8  (-43.4 – -29.4) |
| Non-melanoma skin cancer | 29 | 26 | 14.5  (13.3 – 16.0) | 24.0  (21.9 – 26.5) | 10.6  (9.6 – 11.8) | 30.5  (19.0 – 43.9) | 22.4  (11.6 – 34.9) | -18.8  (-26.6 – -9.8) |
| Nasopharynx cancer | 26 | 27 | 12.9  (11.6 – 14.5) | 21.3  (19.2 – 23.9) | 12.2  (10.9 – 13.7) | -5.4  (-15.3 – 6.1) | -11.3  (-20.6 – -0.6) | -30.6  (-38.2 – -21.6) |
| Hodgkin lymphoma | 25 | 28 | 11.0  (8.9 – 14.6) | 18.1  (14.7 – 24.1) | 13.8  (11.2 – 18.5) | -45.9  (-52.6 – -29.4) | -49.2  (-55.5 – -33.9) | -53.8  (-59.9 – -38.9) |
| Thyroid cancer | 27 | 29 | 10.5  (9.5 – 11.5) | 17.3  (15.6 – 18.9) | 8.6  (7.8 – 9.4) | -14.0  (-22.2 – -5.8) | -19.4  (-27.0 – -11.7) | -40.2  (-45.9 – -34.2) |
| Testicular cancer | 30 | 30 | 3.0  (2.6 – 3.5) | 5.0  (4.3 – 5.8) | 4.8  (4.0 – 5.6) | -30.7  (-42.1 – -18.7) | -35.1  (-45.8 – -23.8) | -31.2  (-43.4 – -18.3) |

UI: uncertainty intervals.

^a^Includes malignant neoplasms (International Classification of Diseases 10 [ICD-10] codes C00-C96, excluding Kaposi sarcoma, ICD-10 C46), benign/in situ neoplasms (ICD-10 D00-D49), and other malignant neoplasms (ICD-10 codes C17, C30-C31, C37, C38, C40-C41, C47-C49, C4A, C51-C52, C57-C58, C60, C63, C66, C68, C69, C74-C75). ^b^Includes other malignant neoplasms (ICD-10 codes C17, C30-C31, C37, C38, C40-C41, C47-C49, C4A, C51-C52, C57-C58, C60, C63, C66, C68, C69, C74-C75). ^c^Includes benign/in situ neoplasms (ICD-10 D00-D49).

**Supplementary Table S6.** Number of years living with disability (YLDs), YLD rates, and age-standardized YLD rates for all cancers and 30 cancer groups, Italy, 2017, and percent change between 1990 and 2017 (ordered by ranking in number of YLDs in 2017).

| **Cancer site** | **Rank by number of YLDs in 2017** | | **YLDs**  **(in Thousands) (95% UI)** | **YLD rate per**  **100 000 person-years (95% UI)** | **Age-standardized YLD rate per**  **100 000 person-years**  **(95% UI)** | **Median change (%)**  **between 1990 and 2017** | | |
| --- | --- | --- | --- | --- | --- | --- | --- | --- |
|  | **1990** | **2017** | **2017** | **2017** | **2017** | **YLDs**  **(95% UI)** | **YLD rate**  **(95% UI)** | **Age-standardized YLD rate**  **(95% UI)** |
| All cancers^a^ |  |  | 192.1  (140.9 – 254.1) | 317.0  (232.6 – 419.3) | 165.0  (120.2 – 220.9) | 68.2  (55.9 – 82.5) | 57.7  (46.1 – 71.1) | 20.2  (11.2 – 30.2) |
| Breast cancer | 1 | 1 | 36.2  (25.0 – 49.7) | 59.7  (41.3 – 82.1) | 32.2  (22.4 – 44.7) | 42.1  (23.5 – 61.2) | 33.2  (15.8 – 51.1) | 1.5  (-12.3 – 16.0) |
| Colon and rectum cancer | 2 | 2 | 26.6  (19.4 – 35.0) | 43.8  (32.0 – 57.7) | 19.8  (14.3 – 26.4) | 80.8  (61.9 – 102.1) | 69.4  (51.8 – 89.4) | 19.6  (6.7 – 34.1) |
| Prostate cancer | 3 | 3 | 26.3  (17.5 – 42.9) | 43.4  (28.9 – 70.7) | 19.3  (12.7 – 31.8) | 126.1  (79.5 – 211.8) | 111.9  (68.3 – 192.3) | 57.3  (23.1 – 119.6) |
| Bladder cancer | 4 | 4 | 12.3  (8.8 – 16.4) | 20.3  (14.6 – 27.1) | 8.9  (6.3 – 11.9) | 27.3  (12.6 – 42.7) | 19.4  (5.5 – 33.8) | -16.7  (-27 – -5.8) |
| Other malignant neoplasms^b^ | 9 | 5 | 10.8  (6.7 – 16.5) | 17.8  (11.0 – 27.3) | 12.5  (7.7 – 19.7) | 268.1  (142.4 – 472.0) | 245.0  (127.2 – 436.2) | 196.8  (94.2 – 368.2) |
| Tracheal, bronchus, and lung cancer | 5 | 6 | 10.3  (7.5 – 13.4) | 17.0  (12.3 – 22.1) | 7.7  (5.6 – 10.1) | 42.6  (19.3 – 70.9) | 33.7  (11.9 – 60.2) | -3.9  (-19.5 – 15.2) |
| Uterine cancer | 18 | 7 | 7.7  (5.2 – 10.6) | 12.6  (8.6 – 17.4) | 6.5  (4.4 – 9.0) | 328.5  (240.4 – 431.9) | 301.6  (219.0 – 398.6) | 207.5  (144.3 – 283.4) |
| Malignant skin melanoma | 13 | 8 | 6.3  (3.3 – 9.3) | 10.4  (5.5 – 15.4) | 6.8  (3.6 – 10.0) | 146.2  (83.1 – 192.6) | 130.8  (71.6 – 174.3) | 91.9  (42.2 – 130.2) |
| Non–Hodgkin lymphoma | 10 | 9 | 6.3  (4.3 – 8.5) | 10.3  (7.2 – 14.0) | 6.0  (4.1 – 8.2) | 114.5  (82.2 – 148.7) | 101.1  (70.8 – 133.1) | 53.9  (29.7 – 78.9) |
| Leukemia | 7 | 10 | 6.2  (4.3 – 8.2) | 10.2  (7.1 – 13.5) | 5.4  (3.8 – 7.2) | 45.2  (14.2 – 83.4) | 36.1  (7.0 – 71.9) | -6.8  (-26.3 – 17.2) |
| Stomach cancer | 6 | 11 | 5.2  (3.8 – 6.8) | 8.5  (6.2 – 11.2) | 3.7  (2.7 – 4.9) | 11.4  (-9.2 – 34.6) | 4.4  (-14.9 – 26.1) | -27.1  (-39.7 – -12.5) |
| Brain and nervous system cancer | 17 | 12 | 4.1  (2.6 – 5.8) | 6.8  (4.3 – 9.6) | 4.8  (3.2 – 6.9) | 124.4  (60.8 – 199.1) | 110.3  (50.7 – 180.4) | 75.3  (30.3 – 141.3) |
| Multiple myeloma | 19 | 13 | 3.8  (2.5 – 5.4) | 6.3  (4.2 – 8.8) | 3.2  (2.1 – 4.4) | 132.3  (72.2 – 211.2) | 117.7  (61.4 – 191.7) | 63.6  (20.8 – 119.6) |
| Kidney cancer | 14 | 14 | 3.7  (2.5 – 5.1) | 6.1  (4.2 – 8.4) | 3.2  (2.2 – 4.5) | 51.8  (26.9 – 81.5) | 42.3  (19.0 – 70.1) | 4.3  (-12.9 – 24.1) |
| Thyroid cancer | 11 | 15 | 3.5  (2.4 – 5.0) | 5.8  (3.9 – 8.2) | 3.5  (2.4 – 5.1) | 24.5  (3.6 – 48.3) | 16.7  (-2.9 – 39.0) | -4.9  (-21.2 – 14.0) |
| Ovarian cancer | 12 | 16 | 3.2  (2.1 – 4.3) | 5.2  (3.5 – 7.2) | 3.1  (2.1 – 4.2) | 20.0  (-6.6 – 55.1) | 12.4  (-12.5 – 45.4) | -10.3  (-31.1 – 16.4) |
| Liver cancer | 16 | 17 | 3.0  (2.1 – 4.1) | 5.0  (3.4 – 6.7) | 2.3  (1.6 – 3.1) | 60.6  (38.1 – 88.0) | 50.5  (29.4 – 76.3) | 11.3  (-4.6 – 30.7) |
| Larynx cancer | 8 | 18 | 2.6  (1.8 – 3.5) | 4.3  (3.0 – 5.8) | 2.2  (1.5 – 2.9) | -11.3  (-28.9 – 9.5) | -16.9  (-33.4 – 2.7) | -36.0  (-48.7 – -21.3) |
| Pancreatic cancer | 20 | 19 | 2.6  (1.7 – 3.4) | 4.2  (2.9 – 5.7) | 1.8  (1.2 – 2.4) | 81.5  (47.7 – 122.3) | 70.1  (38.5 – 108.4) | 15.4  (-3.2 – 38.0) |
| Lip and oral cavity cancer | 15 | 20 | 2.1  (1.5 – 2.8) | 3.5  (2.4 – 4.7) | 1.7  (1.2 – 2.3) | 9.0  (-10.6 – 32.3) | 2.2  (-16.2 – 24.0) | -25.6  (-38.6 – -11.3) |
| Cervical cancer | 21 | 21 | 1.5  (1.0 – 2.1) | 2.5  (1.7 – 3.5) | 1.7  (1.1 – 2.4) | 34.8  (8.8 – 64.4) | 26.4  (2.0 – 54.1) | 7.5  (-15.6 – 34.5) |
| Hodgkin lymphoma | 22 | 22 | 1.4  (0.9 – 2.1) | 2.4  (1.5 – 3.5) | 2.4  (1.5 – 3.7) | 31.2  (-2.8 – 89.7) | 22.9  (-8.9 – 77.8) | 35.1  (-4.3 – 100.2) |
| Gallbladder and biliary tract cancer | 23 | 23 | 1.3  (0.9 – 1.9) | 2.2  (1.4 – 3.1) | 0.9  (0.6 – 1.2) | 45.5  (8.5 – 92.8) | 36.4  (1.7 – 80.7) | -9.2  (-30.8 – 15.8) |
| Testicular cancer | 24 | 24 | 1.3  (0.8 – 1.9) | 2.1  (1.3 – 3.1) | 2.3  (1.4 – 3.5) | 52.6  (7.9 – 109.9) | 43.1  (1.1 – 96.8) | 65.0  (14.2 – 130.1) |
| Other pharynx cancer | 27 | 25 | 0.8  (0.5 – 1.0) | 1.3  (0.9 – 1.7) | 0.7  (0.5 – 0.9) | 37.4  (17.5 – 61.5) | 28.8  (10.1 – 51.4) | 2.6  (-12.5 – 21) |
| Mesothelioma | 26 | 26 | 0.7  (0.5 – 1.0) | 1.2  (0.8 – 1.7) | 0.5  (0.4 – 0.8) | 21.5  (-19.4 – 103.0) | 13.9  (-24.5 – 90.3) | -19.2  (-45.4 – 33.0) |
| Esophageal cancer | 25 | 27 | 0.7  (0.5 – 1.0) | 1.2  (0.8 – 1.6) | 0.6  (0.4 – 0.8) | 9.7  (-5.2 – 27.7) | 2.8  (-11.2 – 19.7) | -25.8  (-35.7 – -13.3) |
| Other neoplasms^c^ | 29 | 28 | 0.6  (0.4 – 0.9) | 1.0  (0.6 – 1.5) | 0.5  (0.3 – 0.7) | 60.9  (29.8 – 103.1) | 50.9  (21.7 – 90.4) | 7.7  (-12.7 – 34.5) |
| Nasopharynx cancer | 28 | 29 | 0.6  (0.4 – 0.8) | 0.9  (0.6 – 1.3) | 0.5  (0.4 – 0.8) | 11.2  (-16.2 – 46.1) | 4.3  (-21.4 – 36.9) | -19.7  (-39.8 – 7.8) |
| Non–melanoma skin cancer | 30 | 30 | 0.5  (0.4 – 0.8) | 0.9  (0.6 – 1.3) | 0.4  (0.3 – 0.6) | 112.8  (90.8 – 139.6) | 99.5  (78.8 – 124.6) | 35.7  (22.3 – 53.0) |

UI: uncertainty intervals.

^a^Includes malignant neoplasms (International Classification of Diseases 10 [ICD-10] codes C00-C96, excluding Kaposi sarcoma, ICD-10 C46), benign/in situ neoplasms (ICD-10 D00-D49), and other malignant neoplasms (ICD-10 codes C17, C30-C31, C37, C38, C40-C41, C47-C49, C4A, C51-C52, C57-C58, C60, C63, C66, C68, C69, C74-C75). ^b^Includes other malignant neoplasms (ICD-10 codes C17, C30-C31, C37, C38, C40-C41, C47-C49, C4A, C51-C52, C57-C58, C60, C63, C66, C68, C69, C74-C75). ^c^Includes benign/in situ neoplasms (ICD-10 D00-D49).
